# Core Fucosylation of Maternal Milk N-Glycan Evokes B Cell Activation by Selectively Promoting the l-Fucose Metabolism of Gut *Bifidobacterium* spp. and *Lactobacillus* spp.

**DOI:** 10.1128/mBio.00128-19

**Published:** 2019-04-02

**Authors:** Ming Li, Yaqiang Bai, Jiaorui Zhou, Wei Huang, Jingyu Yan, Jia Tao, Qingjie Fan, Yang Liu, Di Mei, Qiulong Yan, Jieli Yuan, Patrice Malard, Zhongfu Wang, Jianguo Gu, Naoyuki Tanigchi, Wenzhe Li

**Affiliations:** a College of Basic Medical Science, Dalian Medical University, Dalian, China; b CAS Key Laboratory of Receptor Research, Chinese Academy of Sciences, Shanghai Institute of Materia Medica, Shanghai, China; c Dalian Institute of Chemical Physics, Key Laboratory of Separation Science for Analytical Chemistry, Chinese Academy of Sciences, Dalian, China; d Department of Gynaecology, The First Affiliated Hospital of Jinzhou Medical University, Jinzhou, China; e Clinical Laboratory of Huludao Center Hospital, Huludao, China; f Biostime Institute Nutrition & Care (BINC), Guangzhou, China; g Educational Ministry Key Laboratory of Resource Biology and Biotechnology in Western China, Life Science College, Northwest University, Xian, China; h Division of Regulatory Glycobiology, Institute of Molecular Biomembrane and Glycobiology, Tohoku Medical and Pharmaceutical University, Sendai, Japan; i Department of Glyco-Oncology, Osaka International Cancer Institute, Osaka, Japan; KU Leuven; University of Georgia

**Keywords:** B cells, *Bifidobacterium*, *Lactobacillus*, core fucosylation, infants, milk N-glycan

## Abstract

The maternal milk glycobiome is crucial for shaping the gut microbiota of infants. Although high core fucosylation catalyzed by fucosyltransferase 8 (Fut8) is a general feature of human milk glycoproteins, its role in the formation of a healthy microbiota has not been evaluated. In this study, we found that the core-fucosylated N-glycans in milk of Chinese mothers selectively promoted the colonization of specific gut microbial groups, such as *Bifidobacterium* spp. and *Lactobacillus* spp. in their breast-fed infants during lactation. Compared with *Fut8^+/+^* (WT) mouse-fed neonates, the offspring fed by *Fut8*^+/−^ maternal mice had a distinct gut microbial profile, which was featured by a significant reduction of *Lactobacillus* spp., *Bacteroides* spp., and *Bifidobacterium* spp. and increased abundance of members of the *Lachnospiraceae* NK4A136 group and *Akkermansia* spp. Moreover, these offspring mice showed a lower proportion of splenic CD19^+^ CD69^+^ B lymphocytes and attenuated humoral immune responses upon ovalbumin (OVA) immunization. *In vitro* studies demonstrated that the chemically synthesized core-fucosylated oligosaccharides possessed the ability to promote the growth of tested *Bifidobacterium* and *Lactobacillus* strains in minimal medium. The resulting L-fucose metabolites, lactate and 1,2-propanediol, could promote the activation of B cells via the B cell receptor (BCR)-mediated signaling pathway.

## Abstract (precis)

This study provides novel evidence for the critical role of maternal milk protein glycosylation in shaping early-life gut microbiota and promoting B cell activation of neonates. The special core-fucosylated oligosaccharides might be promising prebiotics for the personalized nutrition of infants.

## INTRODUCTION

The human milk glycobiome plays a pivotal role in shaping the gut microbiota of infants (1, 2). In addition to providing nutrients and energy for newborns (3), glycans in human milk can inhibit the adhesion of pathogens and promote intestinal colonization and growth of probiotics (4, 5), resulting in a *Bifidobacterium* and *Lactobacillus* enriched gut micro-ecosystem in breast-fed infants (6, 7). This early-life microbiome contributes significantly to the health outcomes of infants by regulating the development of their immune system (8, 9). The neonatal innate immune system is biased toward a T_H_2 phenotype, which is associated with allergy and autoimmune diseases, and is biased against T_H_1 cells to avoid harmful proinflammatory responses (10). Aberrant neonatal microbiota composition can elicit abnormal immune responses, which is associated with diseases such as pediatric Crohn’s disease, asthma, and milk allergy during childhood (9). In the newborn, short-chain fatty acids (SCFAs) are produced through the bacterial fermentation of glycans that are present in breast milk and SCFAs increase both mitochondrial energy production and glycolysis, which promote B cell differentiation and antibody (Ab) production (11).

The major glycosylated components in human milk include oligosaccharides (HMOs), glycoproteins, and glycolipids (12). Recent advances in mass spectrometry (MS)-based tools have provided a view of the HMOs’ structures that could be decorated with either fucose and/or sialic acid moieties (13, 14). The fucosylated HMOs were found to function as prebiotics for infant-favorable *Bifidobacterium* strains and vary significantly between mothers of different secretory statuses, which is determined by single nucleotide polymorphisms (SNPs) of the maternal fucosyltransferase 2 (*Fut2*) gene (15). In contrast, despite the high abundance of glycoproteins in milk (approximately 8 g/liter) (12, 13), harboring both N-linked and O-linked glycan moieties, few studies have focused on the regulatory role of milk protein glycosylation on gut microbial structure of neonates, even though bacterium-derived glycohydrolases (GHs) have long been regarded as effective enzymes to release protein-bound glycans (16, 17). Once released from the glycoproteins, these glycans can also be considered selective growth substrates for infant-associated gut microbes (18, 19).

**FIG 1:**
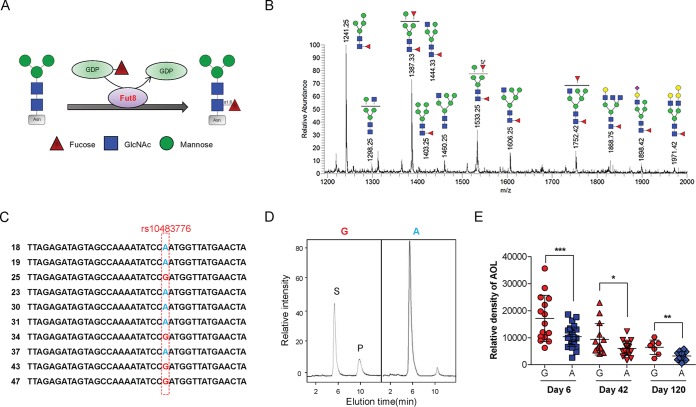
Core fucosylation level of milk N-glycan is associated with maternal *Fut8* gene status. (A) Sketch map of how Fut8 catalyzes the transfer of an L-fucose residue from GDP-fucose to the innermost *N*-acetylglucosamine (GlcNAc) on the asparagine (Asn) residue of N-glycans via α1,6 linkage. (B) Mass spectrometry of N-glycan structures of breast milk protein derived from Chinese mothers. Red triangles, fucose; blue squares, GlcNAc; green circles, mannose; yellow circles, galactose; purple diamonds, NeuAc. (C) The rs10483776 SNP (A→G) allele of the maternal *Fut8* gene detected by DNA sequencing. (D) Fut8 enzyme activities in the cells of breast milk detected by high-performance liquid chromatography (HPLC). Activity was expressed as picomoles of GDP-fucose transferred to the acceptor per hour per milligram of protein. S is the peptide substrate, and P is the product of fucosylation. (E) Densitometric analysis of the bands of AOL in each breast milk sample between the G and A groups at days 6 and 42 postpartum. Data are shown as mean ± SEM (*, *P* < 0.05; **, *P* < 0.01; ***, *P* < 0.001).

Fucosylation is a common type of posttranslational protein modification. Notably, high fucosylation is a general feature of human milk (20), with 75% fucosylation distribution in N-glycans compared with bovine milk (31%). The fucosylation process can be modified by several fucosyltransferases (Fut proteins), including Fut2, Fut3, and Fut8, which transform the fucose residues by α1,2/3, α1,3/4, or α1,6 linkages, respectively (21). For vertebrate protein N-glycans, the main fucosylation process is catalyzed by fucosyltransferase 8 (Fut8), which transfers l-fucose to *N*-acetylglucosamine (GlcNAc) adjacent to the asparagine (N) residue by an α1,6 linkage (Fig. 1A) (22). Fut8-mediated core fucosylation is an important posttranslational process in mammals (23–25). Indeed, the most important and abundant milk proteins, including lactoferrin (LF) and immunoglobulins (IgG, IgM, and secretory IgA [sIgA]) are heavily core fucosylated (21, 26–28). It was found that SNPs in the *Fut8* gene among human populations were closely associated with the fucosylation patterns of N-glycans (29). In addition, our previous studies indicated that *Fut8* gene knockout (*Fut8^−/−^*) mice showed growth retardation and abnormal development of immune system (27, 29–31). We therefore asked whether variations in milk protein core fucosylation levels caused by *Fut8* gene SNP in lactating mothers can affect the gut microbiome of breast-fed infants.

In this study, we first evaluated the correlation between the milk protein core fucosylation levels of Chinese mothers and the gut microbial patterns of their breast-fed infants during lactation. In addition, to investigate the subsequent effects of milk N-glycan core fucosylation status on offspring, we compared the gut microbiota, as well as the immune development of *Fut8^+/+^* mouse neonates fed by *Fut8*^+/−^ or *Fut8*^+/+^ maternal mice. Our study provides novel evidence for the critical role of milk N-glycan in shaping early-life gut microbiota and promoting immune responses.

## RESULTS

### Core fucosylation of milk N-glycan varies significantly among individual mothers.

To investigate the core fucosylation pattern of human milk protein, we isolated whey proteins from the milk samples from 56 Chinese mothers collected at days 6, 42, and 120 postpartum (Table 1). By mass spectrometry, we first profiled the entire milk N-glycan repertoire of Chinese mothers. More than 20 major N-glycan compositions were observed in the milk samples (Fig. 1B). In accordance with previous research (32), the N-glycans in these milk samples were heavily core fucosylated. The high core fucosylation level of milk protein was further demonstrated by lectin blotting with Aspergillus oryzae lectin (AOL), which preferentially recognizes core fucosylation on N-glycans (33). Major milk glycoproteins such as LF, IgA, and IgG are all highly core fucosylated (see Fig. S1A in the supplemental material). Interestingly, we found that the core fucosylation levels of milk proteins varied significantly among individual mothers (Fig. S1B). A previous study showed that the plasma fucosylation levels were highly correlated with SNPs in the human *Fut8* gene (34), in which a G/A polymorphism of rs10483776 on chromosome 14 significantly affected plasma fucosylation levels of females. By DNA sequencing, the A→G polymorphism of rs10483776 was found in 15 of the 56 Chinese mothers providing samples (Fig. 1C; see Table S1 in the supplemental material). Moreover, the Fut8 enzyme activity of the cells in milk samples was increased in mothers within the “G” group (Fig. 1D). Importantly, the core fucosylation level of milk proteins from G group mothers was markedly higher than that of the “A” group mothers during different lactation stages (day 6, *P* = 0.0004; day 42, *P* = 0.0236; and day 120, *P* = 0.0027), while the protein concentrations of milk samples within the two groups were similar (Fig. 1E; see Fig. S2 in the supplemental material [all *P* values are >0.05]).

**TABLE 1:** Subject characteristics

Characteristic	Value for^*a*^:	
	Group G	Group A
*Fut8* gene SNP at rs10483776	G (*n* = 15)	A (*n* = 28)
No. of samples from milk/infants’ feces		
Day 6	15/11	28/13
Day 42	14/11	19/12
Day 120	7/7	13/11
Maternal age (yr)	28.50 ± 0.487	27.85 ± 0.487
Maternal BMI (kg/m^2^)^*b*^	25.48 ± 4.875	25.90 ± 5.625
Gestation duration (wk)	38.86 ± 1.025	38.05 ± 0.928
Gender of infants, % male (no. male/total)	53.33 (8/15)	50.00 (14/28)
Body wt of infants (kg)		
Birth	3.106 ± 0.146	3.098 ± 0.087
Day 6	3.425 ± 0.175	3.405 ± 0.315
Day 42	5.014 ± 0.168	5.003 ± 0.575
Day 120	7.246 ± 0.388	7.180 ± 0.650

a Values are means ± standard deviations (SD), except for numbers or percentages of samples. The total number of values was 56. The total number of samples from secretor mothers was 43. The mode of delivery was vaginal for 100% of the mothers, and the mode of feeding was breastfeeding for 100% of the infants. Maternal secretor status was found to affect the gut microbiota composition of breast-fed infants. We examined the maternal secretor status and eliminated samples of nonsecretor mothers (*n* = 13) and their infants (Table S1). The remaining secretors were grouped into the G and A groups according to their *Fut8* gene status (SNP at rs10483776).

b BMI, body mass index.

10.1128/mBio.00128-19.2 FIG S1 (A) Western blot analysis of breast milk proteins. Plates were incubated with antilactoferrin (anti-LF) Ab (1:2,000), anti-IgA Ab (1:10,000), and anti-IgG Ab (1: 5,000). (B) The fusosylated breast milk proteins analyzed by AOL blotting. Plates were incubated with biotin-conjugated AOL (1:8,000). Download FIG S1, PDF file, 0.1 MB. Copyright © 2019 Li et al. 2019 Li et al. This content is distributed under the terms of the Creative Commons Attribution 4.0 International license.

10.1128/mBio.00128-19.3 FIG S2 (A) Coomassie brilliant blue (CBB) staining of proteins shows comparable amounts of whole-protein lysates in each sample. (B) Detection of breast milk protein concentration by a bicinchoninic acid (BCA) protein assay kit. G, high-core-fucosylation group; A, low-core-fucosylation group. n.s., no significant differences were detected. Download FIG S2, TIF file, 2.2 MB. Copyright © 2019 Li et al. 2019 Li et al. This content is distributed under the terms of the Creative Commons Attribution 4.0 International license.

10.1128/mBio.00128-19.8 TABLE S1 The polymorphism of the *Fut8* gene at rs10483776 and the secretor statuses of different mothers enrolled in this study. Download Table S1, DOCX file, 0.1 MB. Copyright © 2019 Li et al. 2019 Li et al. This content is distributed under the terms of the Creative Commons Attribution 4.0 International license.

### Core fucosylation level of maternal milk N-glycan affects the gut microbiome of infants.

Because the core fucosylation levels of milk N-glycan were significantly different among individual mothers, we investigated whether this variation affects the gut microbiota composition of their breast-fed infants. By high-throughout sequencing of gut bacterial genes, we analyzed the intestinal microbiome of infants at days 6, 42, and 120 after birth (Table 1). Because maternal *Fut2* gene status was found to affect HMO abundance and the gut microbiota composition of breast-fed infants, we examined the maternal secretor status and eliminated samples of nonsecretor mothers and their infants (Table 1; Table S1). Of the remaining samples, a comparison of the total and major HMOs between the A and G groups resulted in no significant difference as detected by a newly developed liquid chromatography-mass spectrometry (LC-MS) system (Fig. S3 [details in Materials and Methods]).

10.1128/mBio.00128-19.4 FIG S3 Comparison of the total and major HMOs between the A and G groups. (A) Statistical analysis of HMOs was performed between the G and A groups at days 6, 42, and 120 postpartum. (B) The composition and structures of HMOs. Data are reported as mean ± SEM. No significant differences between groups were detected. Download FIG S3, TIF file, 0.9 MB. Copyright © 2019 Li et al. 2019 Li et al. This content is distributed under the terms of the Creative Commons Attribution 4.0 International license.

**FIG 2:**
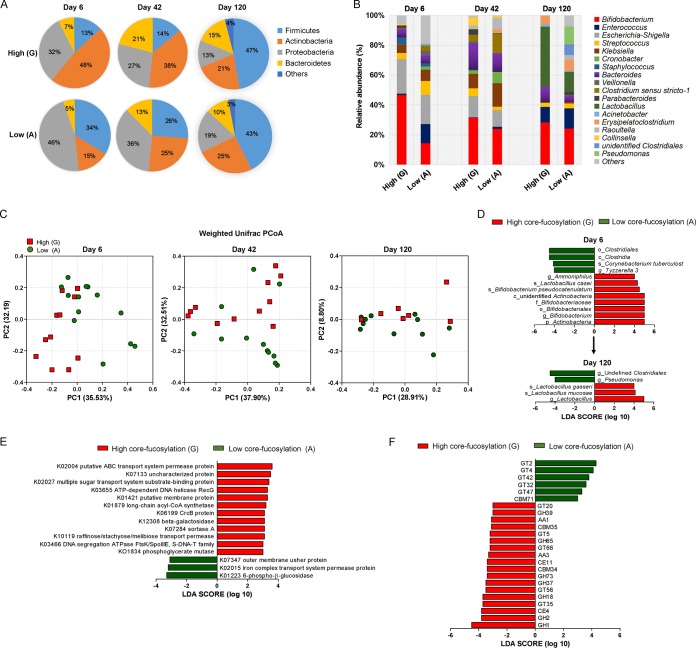
Core fucosylation of milk N-glycan regulates the gut microbiome of infants. (A) The fecal microbiota profiles of infants fed by mothers with high (G group)- or low (A group)-core-fucosylated milk N-glycan at the phylum level based on 16S rRNA gene sequencing. (B) The fecal microbiota profiles of infants at genus level. (C) Characteristics of infant gut microbiota at days 6, 42, and 120 postpartum, illustrated by principal-coordinate analysis (PCoA) clustering analyses. Data from individuals (points) were clustered, and the centers of gravity were computed for each class. (D) The linear discriminant analysis effect size (LefSe) was adopted to identify the bacterial groups that showed significant differences in abundance between the high (G) and low (A) groups at day 6 postpartum. (E) Use of the Kyoto Encyclopedia of Genes and Genomes (KEGG) to evaluate the gut microbial functions between the high and low groups at day 42 postpartum. In order to screen the marker genes with significant differences between groups, the difference function between different groups was first detected by rank sum test and the dimension reduction was evaluated by LDA (linear discriminant analysis). The length of the histogram represents the influence of the difference function. (F) The Carbohydrate-Active enZYmes Database (CAZy) was used to evaluate the gut microbial functions of infants within the high and low groups at day 42 postpartum. GHs, glycoside hydrolases; GTs, glycosyl transferases; PLs, polysaccharide lyases; CEs, carbohydrate esterases; AAs, auxiliary activities; CBMs, carbohydrate-binding modules.

The 16S rRNA sequencing of fecal samples from 6-day-old infants obtained a total of 1,968,299 high-quality filtered reads, which were assembled into 82,012 effective tags per infant. After systematic analysis, we found that the alpha diversity of infants’ gut microbiota reflected by Chao1 and the Shannon index showed no significant difference between the corresponding groups (Table S2 [all *P* values are >0.05). The dominant bacterial phyla in the gut of 6-day-old infants fed by G group mothers were *Actinobacteria* (47.89% ± 15.45%) and *Proteobacteria* (31.57% ± 16.71%), which constituted more than 79.46% of the total bacteria (Fig. 2A). In contrast, the gut microbiota of infants fed by A group mothers was dominated by *Proteobacteria* (46.37% ± 14.20%) and *Firmicutes* (33.73% ± 15.43%), with a lower abundance of *Actinobacteria* (14.55% ± 6.78%; *P* = 0.0438 compared with G group). By day 42, the relative abundances of *Actinobacteria* (37.56% ± 9.87%) and *Proteobacteria* (27.20% ± 7.21%) still occupied the major proportion of total gut microbiota in infants fed by high-G-group mothers, and the average abundance of *Bacteriodetes* in these infants was 20.86% ± 8.22%. In contrast, the abundances of *Actinobacteria* (25.27% ± 3.15%) and *Bacteriodetes* (12.50% ± 7.29%) were much lower in gut of infants fed by A group mothers (*P* = 0.0285 and *P* = 0.0327). By day 120, the relative abundance of *Firmicutes* had become the major phylum in infants of both groups (47.20% ± 8.51% in the G group and 42.89% ± 6.73% in the A group; *P* > 0.05), the abundance of *Actinobacteria* in the G group is 21.30% ± 3.43%, which was no longer higher than that in the A group (25.35% ± 5.57%; *P* > 0.05).

10.1128/mBio.00128-19.9 TABLE S2 Comparison of alpha diversity indexes of infants’ gut microbiota between groups. Download Table S2, DOCX file, 0.1 MB. Copyright © 2019 Li et al. 2019 Li et al. This content is distributed under the terms of the Creative Commons Attribution 4.0 International license.

At the genus level (Fig. 2B), 6-day-old infants fed by mothers with higher core-fucosylated milk (G group) harbored a higher abundance of *Bifidobacterium* (46.68% ± 5.24%), whereas the relative abundance of *Bifidobacterium* (14.29% ± 3.61%) was significantly lower (*P* = 0.0028) in the gut of A group infants. By days 42 and 120, *Bifidobacterium* was still more abundant in infants fed by G group mothers than in those fed by A group mothers (day 42, 31.46% ± 3.47% versus 23.97% ± 2.96%, *P* = 0.0462; day 120, 30.59% ± 3.65% versus 25.36% ± 5.83%, *P* > 0.05). In contrast, the abundance of *Lactobacillus* spp. in gut of infants was much lower compared than that of *Bifidobacterium* spp. during early lactation (day 6 to day 42); *Lactobacillus* occupied no more than 1.00% of the total bacteria. However, the proportion of this genus was largely increased by day 120, and we observed a significant difference between the G and A groups (43.19% ± 7.89% versus 14.21% ± 3.71%; *P* = 0.0256). The major *Bifidobacterium* and *Lactobacillus* spp. detected in gut of the infants include Bifidobacterium pseudocatenulatum, Bifidobacterium bifidum, Bifidobacterium breve, Lactobacillus gasseri, Lactobacillus mucosae, Lactobacillus casei, and Lactobacillus fermentum, etc.: the relative abundances of most of these species were much higher in G group infants than those in A group infants as detected at days 6, 42, and 120 postbirth (see Fig. S4 in the supplemental material).

10.1128/mBio.00128-19.5 FIG S4 (A) Comparison of the abundance of *Bifidobacterium* species detected in gut of infants within low- and high-core-fucosylation groups (groups A and G, respectively). (B) Comparison of the abundance of *Lactobacillus* species detected in gut of infants within groups A and G groups. Data are reported as mean ± SEM. *, *P* < 0.05, **, *P* < 0.01, and ***, *P* < 0.001, by unpaired Student’s *t* test (*n* = 3). Download FIG S4, TIF file, 5.0 MB. Copyright © 2019 Li et al. 2019 Li et al. This content is distributed under the terms of the Creative Commons Attribution 4.0 International license.

By using principal-coordinate analysis (PCoA), we decomposed all the OTU data into two main factors that explained 67.72% (day 6), 70.41% (day 42), and 37.71% (day 120) of the variance (Fig. 2C). All the fecal samples of infants mainly clustered into the two groups were highly correlated with the maternal *Fut8* gene phenotype (day 6, *P* = 0.0026, day 42, *P* = 0.04937, and day 120, *P* = 0.0385, as analyzed by weighted UniFrac *t* test). The linear discriminant analysis (LDA) effect size (LEfSe) was further adopted to identify the bacterial groups that showed significant differences in abundance between groups. As shown in Fig. 2D, the phylum of *Actinobacteria* in infants fed by G group mothers at day 6 was significantly (LDA score = 5.00) more abundant than that for the A group, which mainly contained *B. pseudocatenulatum* (LDA score = 4.48). The genus *Ammoniphilus* and species of *L. casei* were also significantly more abundant in infants within the G group (LDA scores = 4.02 and 4.32, respectively). In contrast, the key phylotypes (significantly more abundant microbial groups) detected in infants fed by mothers with low-core-fucosylated breast milk (A group) was the class of *Clostridia* (LDA score = 4.49), which mainly contained a rich abundance of *Tyzzerella* (LDA score = 4.00), a genus belonging to the family of *Lachnospiraceae* under the order of *Clostridiales*. In addition, Corynebacterium tuberculostearicum, part of the normal skin flora (35), was also abundant in this group (LDA score = 4.11). *Ammoniphilus* and *Corynebacterium* are rarely found in human gut; thus, our observation may due to environmental and ethnic effects on our study cohort. No key phylotypes were detected between infants of the two groups by day 42. However, as the lactation duration increased, the *Lactobacillus* spp. (LDA score = 4.95) became new biomarkers in the gut of infants fed by mothers with high-core-fucosylated milk, and the genera *Pseudomonas* (LDA score = 4.00) and undefined *Clostridiales* (LDA score = 4.47) were significantly more abundant in infants fed by mothers with low-core-fucosylated milk N-glycans.

Using the Kyoto Encyclopedia of Genes and Genomes (KEGG) and the Carbohydrate-Active enZYmes Database (CAZy), we further evaluated the gut microbial functions between the groups to identify the major enzymes/pathways involved in the metabolism of fucosylated milk N-glycans. Fifteen KEGG modules were preferentially enriched in feces of infants collected by day 42 postbirth (Fig. 2E; *n* = 11 in the G group, and *n* = 12 in the A group): in the G group, 12 were identified as β-galactosidase (K12308), putative ABC transport system permease protein (K10119), long-chain acyl coenzyme A (acyl-CoA) synthetase (K01897), implicating the upregulation of glycan hydrolysis, transportation, and biosynthesis of energy in gut microbes. The CAZy database analysis revealed that infants fed by G group mothers had enriched genes associated with glycoside hydrolases (GHs) and glycosyl transferases (GTs) (Fig. 2F).

### *Fut8*^+/−^ maternal mouse-fed neonates have distinct gut microbiota.

**FIG 3:**
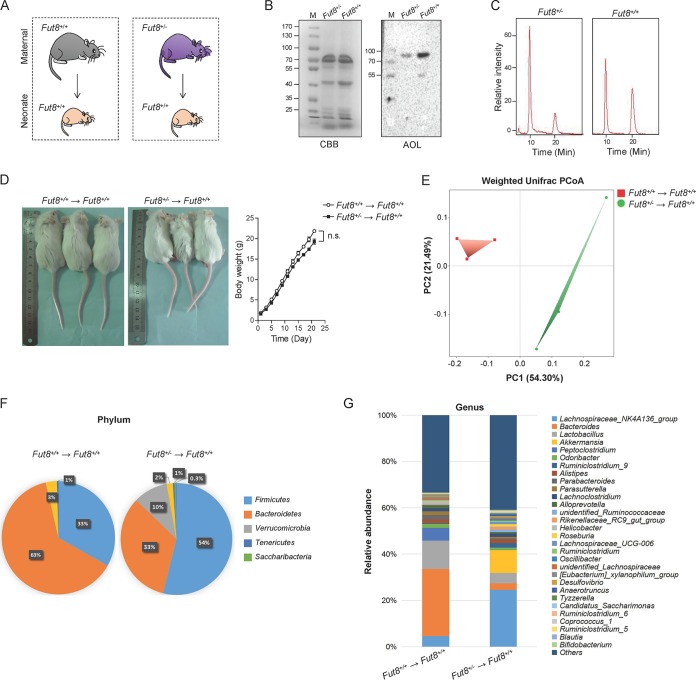
*Fut8*^+/−^ maternal mouse-fed neonates have distinct gut microbiota compared with the *Fut8*^+/+^ maternal mouse-fed neonates. (A) Experimental design. Female mice with a heterozygous *Fut8* gene (*Fut8*^+/−^) were adopted as maternal models with low-core-fucosylated milk N-glycan compared with wild-type mice (*Fut8^+/+^*), and their effects on *Fut8*^+/+^ offspring were evaluated. (B) The breast milk proteins of mice were analyzed by SDS-PAGE and AOL blotting (1:8,000). The Coomassie brilliant blue (CBB) staining of gels shows comparable amounts of whole-protein lysates in each sample. (C) Fut8 enzyme activities in the cells of mammary gland from maternal mice by detected by HPLC. (D) Photos of the neonatal mice fed by *Fut8^+/+^* and *Fut8*^+/−^ mothers after 3 weeks of lactation, and their body weights during lactation were compared. Data are reported as mean ± SEM. n.s., no significant differences between groups were detected. (E) The fecal microbiota profiles of *Fut8^+/+^* and *Fut8*^+/−^ maternal mouse-fed offspring at the phylum level based on 16S rRNA gene sequencing. (F) The PCoA revealed distinct clustering of microbiota composition between *Fut8^+/+^* and *Fut8*^+/−^ maternal mouse-fed offspring. (G) The relative abundance of fecal microbial groups of *Fut8^+/+^* and *Fut8*^+/−^ maternal mouse-fed offspring at the genus level.

To examine the effects of milk N-glycan core fucosylation on offspring gut microbiota and health development, female mice with a heterozygous *Fut8* gene (*Fut8*^+/−^) were adopted as maternal models with low-core-fucosylated milk N-glycan compared with wild-type (*Fut8^+/+^*) mice, and their effects on *Fut8^+/+^* offspring were evaluated (Fig. 3A). As expected, the core fucosylation level of N-glycans in the breast milk of *Fut8*^+/−^ mice was reduced compared with that in *Fut8^+/+^* mice (Fig. 3B). The activity of Fut8 in *Fut8*^+/−^ mice was about half that of *Fut8^+/+^* mice (Fig. 3C). After lactation for 3 weeks, the body weight of offspring mice fed by *Fut8*^+/−^ mice was similar to those of offspring fed by *Fut8^+/+^* mice (Fig. 3D; *P* > 0.05, *n* = 3). However, alterations in the gut microbiota of the offspring fed by *Fut8*^+/−^ mice were observed. As shown in Fig. 3E, the PCoA revealed a distinct clustering of microbiota composition between *Fut8^+/+^* and *Fut8*^+/−^ mouse-fed offspring, suggesting a significant difference in their beta diversity (*P* = 0.0363, weighted UniFrac *t* test). The major bacterial phyla in offspring fed by *Fut8^+/+^* mice were *Bacteroidetes* (63.13% ± 5.85%), *Firmicutes* (33.21% ± 5.32%), and *Proteobacteria* (3.16% ± 0.38%) (Fig. 3F). Notably, the *Bacteroidetes* group (especially Bacteroides acidifaciens), which can ferment a wide range of sugar derivatives (36), was significantly affected by the reduction of core-fucosylated N-glycan in the milk of *Fut8*^+/−^ mice (33.30% ± 6.89%, *P* = 0.0475, compared with *Fut8^+/+^* mouse-fed neonates). Conversely, *Firmicutes* (53.80% ± 9.65%) and *Verrucomicrobia* (9.95% ± 0.85%) were more abundant in the gut of *Fut8*^+/−^ mouse-fed neonates (*P* = 0.0221 and *P* = 0.019, respectively). At the genus level, the gut of neonates fed by *Fut8*^+/−^ mice was characterized by reduction of *Bacteroides* (29.02% ± 1.20% versus 2.75% ± 0.08%; *P* = 0.0360) and *Lactobacillus* (12.25% ± 1.21% versus 4.52% ± 0.49%; *P* = 0.0389) and increased abundance of members of the *Lachnospiraceae* NK4A136 group (4.61% ± 0.48% versus 24.63% ± 1.59%; *P* = 0.0152) and *Akkermansia* spp. (0.03% ± 0.00% versus 9.94% ± 0.85%; *P* = 0.0179), compared with *Fut8^+/+^* mouse-fed neonates (Fig. 3G). Bifidobacteria are not autochthonous to the mouse gut, but we still detected a small portion of them in mice of our study: the abundance of *Bifidobacterium* was found significantly lower in the neonates fed by *Fut8*^+/−^ mice than in those fed by *Fut8^+/+^* mice (0.22% ± 0.08% versus 0.02% ± 0.01%; *P* = 0.0099) (see Fig. S5 in the supplemental material).

10.1128/mBio.00128-19.6 FIG S5 Comparison of the abundance of gut *Bifidobacterium* spp. between *Fut8^+/+^* and *Fut8*^+/−^ maternal mouse-fed offspring. Data are reported as mean ± SEM. *P* = 0.0099, unpaired Student’s *t* test (*n* = 3). Download FIG S5, TIF file, 0.7 MB. Copyright © 2019 Li et al. 2019 Li et al. This content is distributed under the terms of the Creative Commons Attribution 4.0 International license.

### *Fut8*^+/−^ maternal mouse-fed neonates showed lower proportion of splenic CD19^+^ CD69^+^ B lymphocytes and attenuated humoral immune response.

**FIG 4:**
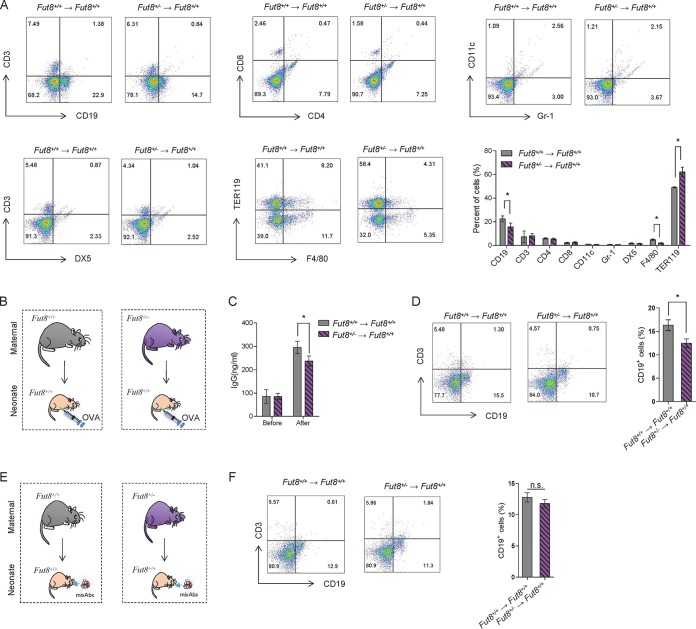
*Fut8*^+/−^ maternal mouse-fed neonates showed a lower proportion of splenic CD19^+^ CD69^+^ B lymphocytes and attenuated humoral immune response. (A) Flow cytometry analysis of the proportion of spleen cells in *Fut8^+/+^* and *Fut8*^+/−^ maternal mouse-fed offspring. Numbers indicate the percentage of the indicated cell groups in total spleen cells. Ten thousand events were acquired for each analysis. Data are representative of three independent experiments and are shown as mean ± SEM (*, *P* < 0.05). (B) Experimental design. *Fut8^+/+^* and *Fut8*^+/−^ maternal mouse-fed offspring were immunized by abdominal cavity injection with 50 µg of OVA mixed with an equal volume of complete Freund’s adjuvant (CFA). Two weeks later, mice were immunized with 50 µg of OVA by subcutaneous injection. Mice sera were collected before and 14 days post-OVA immunization. (C) Comparison of the levels of serum IgG in *Fut8^+/+^* and *Fut8*^+/−^ maternal mouse-fed offspring after immunization. The concentrations of IgG in the sera of mice (*n* = 3/group) were measured by enzyme-linked immunosorbent assay (ELISA) using mouse MAb isotyping reagents. Data are shown as mean ± SEM (*n* = 3; *, *P* < 0.05). (D) Flow cytometry analysis of the CD3^+^ and CD19^+^ cell proportions in spleen of *Fut8^+/+^* and *Fut8*^+/−^ maternal mouse-fed offspring after immunization. Data are shown as mean ± SEM (*n* = 3; *, *P* < 0.05). (E) Experimental design. *Fut8^+/+^* and *Fut8*^+/−^ maternal mouse-fed offspring were orally administered with a minimally effective dose of antibiotics (mixAbx: ampicillin, neomycin, and metronidazole at 40 mg/liter and vancomycin at 20 mg/liter) from the 10th day to the 21st day after birth. (F) Flow cytometry analysis of the CD3^+^ and CD19^+^ cell proportions in spleen of *Fut8^+/+^* and *Fut8*^+/−^ maternal mouse-fed offspring after immunization. Data are shown as mean ± SEM (*n* = 3). n.s., no significant differences were detected between groups.

Since aberrant neonatal microbiota composition can elicit abnormal immune responses, we further investigated the population of lymphocytes in spleen and thymus of offspring mice. A significantly reduction in the total lymphocyte numbers in the spleen and thymus of *Fut8*^+/−^ mouse-fed offspring were detected compared with those fed by *Fut8^+/+^* mice (see Fig. S6 in the supplemental material [*P* = 0.0076 and *P* = 0.0009; *n* = 3]). Flow cytometry analysis revealed that the frequencies of B cells (CD19^+^) and macrophages (F4/80^+^) were significantly reduced in *Fut8*^+/−^ mouse-fed offspring (*P* = 0.0454 and *P* = 0.0233; *n* = 3), whereas those of TER119^+^ cells were increased (Fig. 4A [*P* = 0.0439; *n* = 3]), The proportions of CD4^+^ T cells, CD8^+^ T cells, Gr-1^+^ cells, CD11C^+^ cells, and NK cells (DX5^+^) in these mice were comparable with control. As B cells play important roles in humoral immunity by secreting antibodies, we therefore immunized the neonates after breastfeeding for 3 weeks by *Fut8*^+/−^ or *Fut8^+/+^* mice (Fig. 4B) and compared the serum IgG levels of them to each other. As shown in Fig. 4C, significantly lower level of serum IgG in *Fut8*^+/−^ mouse-fed offspring (*P* = 0.0421; *n* = 3) was found compared with *Fut8^+/+^* mouse-fed offspring post-ovalbumin (OVA) immunization, which was in accordance with the lower proportion of B cells observed (Fig. 4D [*P* = 0.0168; *n* = 3]). However, when we treated the offspring mice with a minimally effective dose of mixed antibiotics (mixAbx), which effectively decreases gut microbiota without affecting B cell survival *in vitro* (Fig. 4E) (11), no difference in splenic B cell proportions was observed between *Fut8^+/+^* and *Fut8*^+/−^ mouse-fed offspring (Fig. 4F [*P* > 0.05]). These results suggested that the regulatory role of core-fucosylated milk N-glycan on humoral immunity of neonates was gut microbiota dependent.

10.1128/mBio.00128-19.7 FIG S6 Comparison of the total lymphocyte numbers in spleen and thymus of neonatal mice fed by *Fu8^+/+^* and *Fut8*^+/−^ maternal mice. Data are reported as mean ± SEM. **, *P* < 0.01, and ***, *P* < 0.001, by unpaired Student’s *t* test (*n* = 3). Download FIG S6, TIF file, 0.1 MB. Copyright © 2019 Li et al. 2019 Li et al. This content is distributed under the terms of the Creative Commons Attribution 4.0 International license.

### Metabolites of l-fucose by *Lactobacillus* and *Bifidobacterium* spp. evoked B cell activation *in vitro*.

**FIG 5:**
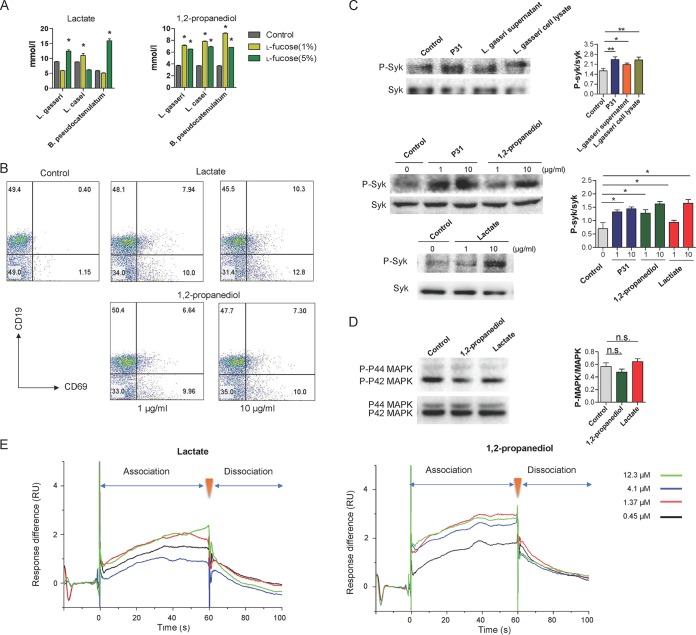
Metabolites of l-fucose by *Lactobacillus* and *Bifidobacterium* evoke B cell activation *in vitro.* (A) The bacterial strains were first cultured in enrichment media (Table S3), and to observe the effects of l-fucose on their growth, they were inoculated in media without glucose and replaced by l-fucose at the indicated final concentrations. The strains cultured in sugar-free media were used as controls. The production of 1,2-propanediol and lactate in culture broth was measured by ELISA. Data are shown as mean ± SEM (*n* = 3). *, *P* < 0.05 compared with the control group. (B) Flow cytometry analysis of the proportion of B cells (CD19^+^ CD69^+^) activated by 1,2-propanediol and lactate in Peyer's patches (PPs) of mice. (C) The phosphorylation level of Syk in 3-83 B cells was assessed upon treatment of l-fucose metabolites. The blots were probed by anti-pSyk and anti-Syk Abs. Shown is densitometric analysis of the bands of pSyk normalized with Syk. Data are shown as mean ± SEM (*n* = 3). *, *P* < 0.05, and **, *P* < 0.01, compared with the control group. (D) The phosphorylation level of p42 and p44 isoforms of MAPK (Erk1 and Erk2, respectively) in 3-83 B cells was assessed. Shown is densitometric analysis of the bands of p42 and p44 MAPK normalized with MAPK. Data are shown as mean ± SEM (*n* = 3). n.s., not significant. (E) The interactions of BCR with 1,2-propanediol and lactate were analyzed by Biacore. Analytic concentrations (from top to bottom) are 12.3, 4.1, 1.37, and 0.45 μM.

Previous studies (37, 38) showed that the metabolism of l-fucose in *Bifidobacterium* and *Lactobacillus* spp. harboring a l-fucose operon resulted in the production of 1,2-propanediol and l-lactate. Indeed, when we cultured *B. pseudocatenulatum* CGMCC1.5001, *L. casei* ATCC 334, and *L. gasseri* ATCC 33323 strains with l-fucose, we detected the growth of these strains and the production of 1,2-propanediol and lactate (Fig. 5A). Next, we asked whether the metabolites of l-fucose can exert effects on B lymphocytes in the Peyer’s patches (PPs) of mice. To test this, we isolated total lymphocytes from the PPs of mice and incubated them with 1,2-propanediol and lactate. The results showed that the frequencies of B cells (CD19^+^) and activated B cells (CD69^+^ CD19^+^) (39) in PPs were markedly increased following stimulation with these metabolites (Fig. 5B [all *P* values are <0.0001; *n* = 3]).

10.1128/mBio.00128-19.10 TABLE S3 The bacterial strains used in this study. Download Table S3, DOCX file, 0.1 MB. Copyright © 2019 Li et al. 2019 Li et al. This content is distributed under the terms of the Creative Commons Attribution 4.0 International license.

The recognition of antigen by the B cell receptor (BCR) complex on the surface of B cells triggers signaling cascades via spleen tyrosine kinase (Syk) that ultimately lead to B cell activation and development (40). To further elucidate the role of l-fucose metabolites in B cell activation, we incubated 1,2-propanediol and lactate with 3-83 B cells (expressing IgG2a-BCR recognizing p31), and assessed the phosphorylation level of Syk. Compared with 3-83 cells, the phosphorylation levels of Syk, as can be induced by p31, were upregulated upon treatment with L. gasseri supernatant and cell lysates (Fig. 5C [all *P* values are <0.05]). Consistent with the change in B cell activation among PP cells, the l-fucose metabolites of L. gasseri enhanced the phosphorylation of Syk at a dose of 10 μg/ml. However, the incubation of 3-83 B cells with 1,2-propanediol or lactate did not result in the increased phosphorylation of the p42 and p44 isoforms of mitogen-activated protein kinase (MAPK) (Erk1 and Erk2, respectively) (Fig. 5D). This suggests that these metabolites activate B cells through BCR signaling pathway, but not MAPK pathway. Furthermore, we adopted a new Biacore strategy to analyze the molecular interactions between L-fucose metabolites and BCR. 1,2-Propanediol and lactate were found to interact directly with BCR protein at a concentration range of 1.37 to ∼12.3 µM (Fig. 5E), which further supported our hypothesis that the l-fucose metabolites of *Bifidobacterium* and *Lactobacillus* spp. induce B cell proliferation and activation through the BCR-mediated signaling pathway.

### Core-fucosylated oligosaccharides promoted the growth of *Bifidobacterium* and *Lactobacillus* spp.

**FIG 6:**
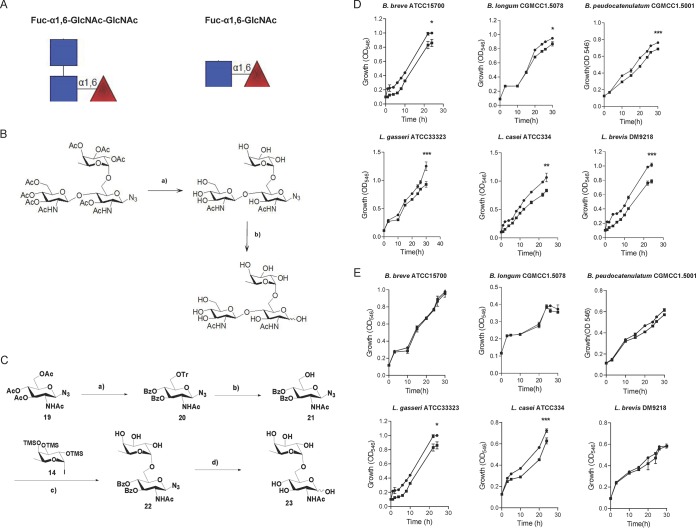
Core-fucosylated oligosaccharides promote the growth of tested *Bifidobacterium* and *Lactobacillus* strains (A) The basic fucosyl structure on milk N-glycan: Fuc-α1,6-GlcNAc-GlcNAc and Fuc-α1,6-GlcNAc. (B) The synthetic process of Fuc-α1,6-GlcNAc-GlcNAc (35). (C) The synthetic process of Fuc-α1,6-GlcNAc (details in Materials and Methods). (D) The growth curves of *Bifidobacterium* and *Lactobacillus* strains when cultured in liquid media supplemented with chemically synthesized Fuc-α1,6-GlcNAc-GlcNAc as the sole carbon source. (E) The growth curves of *Bifidobacterium* and *Lactobacillus* strains when cultured in liquid media supplemented with chemically synthesized Fuc-α1,6-GlcNAc as the sole carbon source. The oligosaccharide concentration was 10 mM. Growth was monitored by measuring the OD_546_. Three technical replicates were performed for each strain. *, *P* < 0.05, **, *P* < 0.01, and ***, *P* < 0.001, by two-way ANOVA.

*Lactobacillus* and *Bifidobacterium* spp. were more abundant in high-core-fucosylated milk-fed infants and *Fut8^+/+^* mouse-fed offspring, suggesting that these bacteria might preferentially use core-fucosylated milk N-glycans. Fuc-α1,6-GlcNAc-GlcNAc and Fuc-α1,6-GlcNAc are basic fucosyl structures on milk N-glycan (Fig. 6A). Therefore, we chemically synthesized Fuc-α1,6-GlcNAc-GlcNAc and Fuc-α1,6-GlcNAc (41) (Fig. 6B and C), and cultured several *Bifidobacterium* and *Lactobacillus* strains (see Table S3 in the supplemental material) in liquid minimal medium supplemented with Fuc-α1,6-GlcNAc and Fuc-α1,6-GlcNAc-GlcNAc as the sole carbon source. Interestingly, most bacteria grew in the Fuc-α1,6-GlcNAc-GlcNAc-supplemented medium (Fig. 6D), but only some of the *Lactobacillus* spp. (*L. casei* ATCC 334 and *L. gasseri* ATCC 33323) survived in Fuc-α1,6-GlcNAc-supplemented medium (Fig. 6E). These results suggest that *Lactobacillus* strains have superior ability to hydrolyze the α1,6-linkage-bond fucose.

## DISCUSSION

Human milk glycans provide a broad range of carbon sources for gut microbes in infants. In addition to HMOs, there are numerous N-glycans in human milk proteins, which may impact the gut microbial composition of infants. Core fucosylation of N-glycan is the most common posttranscriptional modification of proteins in mammals. To our knowledge, this is the first study to systematically investigate the correlation between maternal milk glycosylation and the gut microbiome of infants.

Fucosylated glycans have marked effects on human health through manipulation of gut microbiota (42, 43). Many studies have found that the status of mothers’ secretor gene *Fut2* or Lewis blood-type-related gene *Fut3* could influence the α1,2/3- or α1,3/4-fucosylation levels of HMOs and in turn affect the gut microbiota of their breast-fed infants (15). Although the fucosylation of N-glycans in milk is also modified by Fut2 and Fut3, but according to the glycoforms of human milk proteins detected by multiple reaction monitoring (32), core fucosylation is the major fucosylation form of human milk N-glycans, this was also proved by our mass spectrometry detection in milk samples of Chinese mothers. By lectin blotting of milk protein, we found that the *Fut8* gene SNP (A→G mutation at rs10483776) of the enrolled Chinese mothers strongly affected the fucosylation level of their milk N-glycans. On the other hand, when measuring HMOs in these milk samples, we did not find any difference in the abundances of total and major HMOs between groups A and G, which suggested that the A→G mutation in the *Fut8* gene did not affect the fucosylation levels of HMOs. It is not a surprise that the fucosylation of HMOs is mainly catalyzed by Fut2 and Fut3, while the α1,6-fucosylated glycotopes on milk glycoproteins (catalyzed by Fut8) were found to be absent on HMOs (44, 45): thus, the differences in the gut microbiomes observed in infants fed by the G and A groups of mothers were attributed to the different core fucosylation levels of milk N-glycans.

In infants fed by mothers with high-fucosylated milk N-glycans, the *Bifidobacteria*, mainly including *B. pseudocatenulatum*, *B. bifidum*, and *B. breve*, were dominant during early lactation, which markedly contributed to the high proportion of *Actinobacteria* in infants that possess the ability to use fucosylated glycans (46). These *Bifidobacterium* spp. can be shared by mothers and their offspring as a source of probiotics (47, 48). Furthermore, they have antagonistic activities on Gram-negative enteric pathogens (49) and can improve the inflammatory status of insulin-resistant obese children (50). Other more abundant microbial groups in infants fed by G group mothers, especially during late lactation, included *L. casei*, *L. mucosae*, and *L. gasseri*, which are usually isolated from infant feces (51, 52). *L. casei* possesses a strong ability to ferment milk glycans by the expression of various GHs and gene clusters (53, 54). This strain also shows many beneficial effects on infants such as growth promotion (55) and inhibition of pathogens (56, 57). *L. mucosae* strains have been shown to decrease epithelial permeability and improve epithelial barrier function. The presence of this organism provides competitive exclusion against many pathogenic organisms and help with the development of new probiotic food products (58). L. gasseri was found attenuated allergic airway inflammation through PPARγ (peroxisome proliferator-activated receptor gamma) activation in dendritic cells (59). On the contrary, the gut of infants fed by mothers with low-fucosylated-milk N-glycans were dominated by the order *Clostridiales* (at day 6) or the genera of undefined *Clostridiales* and *Pseudomonas* (at day 120). *Pseudomonas* can be an oral and enteric pathogen found dominant in preterm infants (60); overgrowth of these bacteria may increase the risk of infection under immunocompromised conditions (61). *Clostridiales* are in high prevalence in the first 2 weeks of life (62): this was unfortunately known for a few pathogenic species that include Clostridium botulinum, Clostridium perfringens, Clostridium tetani, and Clostridium difficile in the family *Clostridiaceae* (63). However, in our study, *Clostridiales* were largely overlooked because of the difficulties to culture the organisms *in vitro*. Further understanding of the selective nutritional requirement that favor the growth of these bacteria would be helpful to study their effects on intestinal maturation and health outcomes in infants.

Mouse models can complement human studies and provide further insights into how core fucosylation of milk N-glycans influences the gut microbiota composition and the health outcome of breast-fed infants. Interestingly, a greater abundance of *Lactobacillus* spp. (mainly *L. gasseri* and *L. reuteri*), *Bacteroides* spp., and *Bifidobacterium* spp. was detected in the neonates fed by *Fut8^+/+^* mice compared with those fed by *Fut8*^+/−^ mice. *Bacteroides* spp. are glycan consumers (36): thus, our results also suggested their ability to utilize fucosylated milk N-glycans. In contrast, the *Fut8*^+/−^ mouse-fed neonates had more abundance of members of the *Lachnospiraceae* NK4A136 group and *Akkermansia* spp. than those fed by *Fut8^+/+^* mice. *Lachnospiraceae* abundance in gut of infants was found to be significantly associated with higher body mass index (BMI) and with increased odds of being overweight or obesity (64). However, the presence of the *Lachnospiraceae* NK4A136 group was negatively correlated with intestinal inflammation (65). Thus, the functions of this group of bacteria deserve further investigation. *Akkermansia*, especially the species Akkermansia muciniphila, is known as an intestinal mucin-degrading bacterium (66). Although positive correlations were observed between fucosylated HMOs and *A. muciniphila* (67), this genus was also found to be significantly enriched in infants with eczema (68). It was speculated that higher abundance of *A. muciniphila* in eczematous infants might reduce the integrity of intestinal barrier function and therefore increase the risk of developing eczema. Based on the above information, our results found in the neonatal mice suggested an alteration of gut microbiota toward a probably unhealthy pattern in response to changes in core fucosylation levels of maternal milk N-glycans.

As the aberrant neonatal microbiota composition can elicit abnormal immune responses, we therefore further investigated the major immune organs of the neonates fed by *Fut8^+/+^* or *Fut8*^+/−^ mice. Indeed, a significantly smaller population of total lymphocytes in the thymus and spleen of neonates fed by *Fut8*^+/−^ mice was detected compared with those fed by *Fut8^+/+^* mice. These neonates also exhibited a significantly lower proportion of active B cells in their spleen, resulted in attenuated IgG production after OVA stimulation. Of concern, since microbiota suppression by MixAbx treatment in the offspring mice hindered the development of B cells, regardless of whether the offspring were fed by *Fut8^+/+^* or *Fut8*^+/−^ mice, it is reasonable to think that the core-fucosylated milk N-glycan may regulate neonatal B cell responses through gut microbiota modulation.

Kim et al. (11) reported that gut microbiota-derived SCFAs activated B cells by increasing acetyl-CoA and regulating metabolic sensors to increase oxidative phosphorylation, glycolysis, and fatty acid synthesis, which produce energy and factors required for antibody production, but how B cells interact or import these factors remain unclear. Interestingly, in our study, stimulation of 1,2-propanediol and lactate, the l-fucose metabolites of *B. pseudocatenulatum*, *L. casei*, and *L. gasseri*, to mouse PP-derived lymphocytes resulted in a marked elevation of B cell activation. These suggested a novel way of probiotic bacteria in immune regulation other than through the production of SCFAs. BCR-mediated immune responses to antigen (Ag) stimulation regulate several biological functions, including B cell activation and differentiation. To further study the mechanisms, the 3-83 B cell line was adopted. We found that 1,2-propanediol and lactate could initiate the BCR signaling of 3-83 B cells, which plays an important role in the maturation and survival of B cell lineages, and the consequential humoral immune responses (69). This explains the phenomena we observed in *Fut8*^+/−^ mouse-fed neonates, where the relatively reduced B cell proportion resulted in the downregulation of humoral immunity post-OVA stimulation. Our study thus highlighted a novel mechanism whereby l-fucose metabolites, mainly 1,2-propanediol (37, 38), interacted directly with BCR molecules to promote B cell activation. Further molecular studies regarding the interaction between 1,2-propanediol and BCR may contribute to the elucidation of the underlying mechanisms.

To elucidate how core fucose on milk N-glycan selectively promotes the growth of *Bifidobacterium* and *Lactobacillus* spp., Fuc-α1,6-GlcNAc and Fuc-α1,6-GlcNAc-GlcNAc were synthesized chemically. Fucosylated oligosaccharide is an important core structure that forms part of human mucosal and milk glyco-complexes (70–72). The distinctive α1,6 linkage of fucose on N-GlcNAc may contribute to its selective effects. Previous studies showed that Lactobacillus casei fermented the N-GlcNAc moiety of Fuc-α1,6-GlcNAc and excreted l-fucose (73). It harbors a novel α-l-fucosidase (AflC) gene which specifically hydrolyzes natural α1,6-linked fucosyl-oligosaccharides *in vitro* (74). This might explain why *L. casei* is more abundant in infants fed by mothers with high-core-fucosylated milk N-glycan. However, compared with oligosaccharides, the structure of protein N-glycans is more complicated. Bacterium-derived fucosidases were reported to have very low activity in decomposing the α-1,6-bond core fucose (75). Fuc-α1,6-GlcNAc can only be hydrolyzed by α-l-fucosidases with the assistance of endo-*N*-acetylglucosidases (Endo), which hydrolyzes the N-glycans between two adjacent N-GlcNAc. Some bifidobacteria express Endo-like enzymes, which allows them to become dominant in the intestines of infants by using milk N-glycans (18). Endos mainly belong to the GH18 and GH85 families; they are also found in the genomes of other gut microbes, such as multiple species of *Bacteroides* (accession no. WP_048696603.1; GI no. 880967032), Enterococcus faecalis (accession no.: AAO82555.1; GI no. 29344798), and *Lactobacillus* spp. (accession no. YP_004888239.1; GI no. 1061453). Indeed, we found that GH18 genes were upregulated in the gut of infants fed by mothers with high-core-fucosylated-milk N-glycans (Fig. 2E). Thus, use of synthetic Fuc-α1,6-GlcNAc and Fuc-α1,6-GlcNAc-GlcNAc by *L. casei* and *L. gasseri* suggests the coexpression of Endo and AflC-like enzymes in their genomes and their superiority when competing with other gut microbes. However, when Fuc-α1,6-GlcNAc was incubated with *Bifidobacterium* spp., no obvious growth-promoting effects were detected, although α-l-fucosidase genes were also found in the genomes of *Bifidobacterium* spp., but with poor activity for hydrolyzing the α1,6-linkage-bond fucose (75). Therefore, the substantially greater proportion of bifidobacteria in infants fed by mothers with high-core-fucosylated milk N-glycan may due to excreted l-fucose by *Lactobacillus* spp. This suggests a synergism between *Lactobacillus* and *Bifidobacterium* in digesting milk N-glycans in the gut of infants. However, more studies are needed to define in detail the mechanism how *Lactobacillus* and *Bifidobacterium* specifically consume core-fucosylated N-glycan and impact immune development of infants.

### Conclusions.

**FIG 7:**
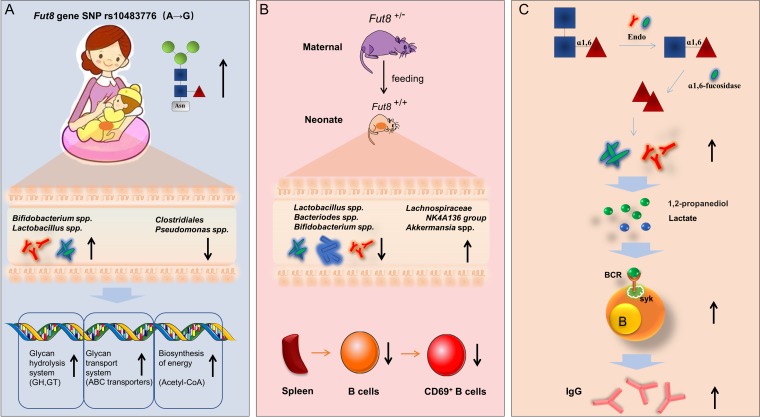
Highlights of this study. (A) Infants fed by mothers with higher-core-fucosylated milk N-glycans (affected by *Fut8* gene status) harbored more abundance of *Bifidobacterium* and *Lactobacillus* spp. and reduced abundance of *Clostridiales* and *Pseudomonas* spp. during lactation. (B) The neonates fed by *Fut8*^+/−^ mice were characterized by reduced abundance of *Lactobacillus*, *Bacteriodes*, and *Bifidobacterium* spp. and increased abundance of members of the *Lachnospiraceae* NK4A136 group and *Akkermansia* spp. in their gut; these neonates also exhibited a lower proportion of active B cells in spleen. (C) *Bifidobacterium* and *Lactobacillus* strains can utilize chemically synthesized core-fucosylated oligosaccharides. Genes encoding Endo and α1,6-fucosidase in the genome of these bacteria are key genetic factors for the use of core fucose, and the resulting metabolites, 1,2-propanediol and lactate, promoted the activation of B cells via the BCR-mediated signaling pathway.

Infants fed by mothers with higher-core-fucosylated milk N-glycans (G group) harbored greater abundance of *Bifidobacterium* spp. and *Lactobacillus* spp. and reduced abundance of *Clostridiales* and *Pseudomonas* during lactation. Compared with *Fut8^+/+^* mouse-fed neonates, the neonates fed by *Fut8*^+/−^ mice were characterized by reduced abundance of *Lactobacillus* spp., *Bacteriodes* spp., and *Bifidobacterium* spp. and increased abundance of members of the *Lachnospiraceae* NK4A136 group and *Akkermansia* spp. in their gut: these neonates also exhibited a lower proportion of active B cells in spleen. I*n vitro* study showed that 1,2-propanediol and lactate, the metabolites of l-fucose produced by *Lactobacillus* and *Bifidobacterium* spp., could evoke B cell activation through the BCR-mediated signaling pathway. The chemically synthesized core-fucosylated oligosaccharides showed the ability to promote the growth of *Lactobacillus* and *Bifidobacterium* spp., and therefore could be considered a promising prebiotic for the personalized nutrition of infants (Fig. 7).

## MATERIALS AND METHODS

### Subjects and sample collection.

The study was approved by the ethical committees of Dalian Medical University, Dalian, China. A subset of 56 infant/mother dyads from the First Affiliated Hospital of Jinzhou Medical University was selected. Written informed consent was obtained from the parents before enrollment. Subjects were enrolled at approximately 34 weeks of gestation and asked to fill out detailed health history questionnaires. To eliminate the interference of delivery mode and feeding pattern on gut microbiome of infants, only mothers who gave birth by vaginal delivery and exclusively breast-fed were enrolled (Table 1). The milk and fecal samples were collected as described before (15) and immediately stored at −20°C until they were transported to the laboratory on dry ice, where they were stored at −80°C prior to use. The milk and feces samples were collected by days 6, 42, and 120 postpartum. Normally 3 days postpartum, the mothers start to lactate, and by day 6, their babies had been fed for at least 3 days; in addition, by that time the mothers have enough milk for sampling. Day 42 is after puerperium, which is the period of adjustment after delivery when the anatomic and physiological changes of pregnancy are reversed and the body returns to the normal nonpregnant state; normally Chinese mothers are asked to go back to the hospital for routine checkup. In addition, according to a previous study (76), from week 4 to week 8 after birth, some microbial groups in the gut of infants significantly increase in proportion: day 42 (6 weeks postpartum) is in the middle of this period. We collected the samples by day 120 postpartum to detect the long-term development of gut microbiota in infants and also to ensure the infants were exclusively breastfeeding, because many of the infants were introduced to formula or complementary foods after 4 months postpartum. All of the infants consumed breast milk only, and infants who received antibiotics, probiotics, or formula powder because of diseases or lack of breast milk were excluded during the study period.

### Detection of milk N-glycan and HMOs by mass spectrometer.

The milk N-glycan detection was carried out using an LTQ-XL linear ion trap electrospray ionization mass spectrometer (ESI-MS) coupled with a high-performance liquid chromatography (HPLC) system (Thermo Scientific, USA), as described previously (77). The HMOs were detected by a newly developed method based on a zwitterionic LC matrix with a mixed-mode action of hydrophilic interaction with cation exchange for cleanup and separation of HMOs (13, 78). The neutral sugars were eluted, and acidic HMOs were resolved and identified. Particularly, we used a different column material (named ASP; 150 mm by 2.1-mm inside diameter [i.d.]) to allow acidic sugars to be eluted first, and focused on detailed separation and neutral HMOs. HMO identification and quantification were performed via Agilent Mass Hunter Qualitative Analysis software (version B.03.01) (detailed in Text S1 in the supplemental material).

10.1128/mBio.00128-19.1 TEXT S1 Supplemental materials and methods describing HMO extraction and detection, fecal DNA extraction, PCR and 16S rRNA amplicon data processing, and metagenomic sequencing and gene catalogue construction. Download Text S1, DOCX file, 0.1 MB. Copyright © 2019 Li et al. 2019 Li et al. This content is distributed under the terms of the Creative Commons Attribution 4.0 International license.

### SNPs.

DNA was extracted from breast milk using the Qiagen DNeasy blood and tissue kit (Qiagen, Venlo, The Netherlands). Genomic DNA purified from each mother’s breast milk was amplified with primers *Fut8*-F (5′-TAT AAA GGC ACA GAA ACA GAC A) and *Fut8*-R (5′-TTG ATG GTG GCT CCA TTG CC), which produces a 337-bp amplicon containing the mutated rs10483776 SNP (A→G) allele of the *Fut8* gene. Successful amplification was confirmed by gel electrophoresis, and the PCR products were sent to the sequencing company (Sangon Biotech) for DNA sequence detection.

### FUT8 enzyme activity assay.

Cells isolated from human milk and mouse mammary gland were suspended in 20 μl lysis buffer containing 10 mM Tris-HCl (pH 7.4), 150 mM NaCl, and 1% Triton X-100. The cell lysate was assayed for Fut8 activity by HPLC, as described previously (23).

### Western blot and lectin blot analysis.

Each milk sample was defatted via centrifugation at 8,000 rpm for 10 min. After that, 1 μl of skim milk was taken and dissolved in 30 μl protein loading buffer (250 mM Tris-HCl [pH 6.8], 0.5% bromophenol blue, 50% glycerol, 10% SDS, 5% β-mercaptoethanol), and then 10 μl of proteins was subjected to SDS-PAGE. After SDS-PAGE, the proteins were transferred to polyvinylidene difluoride (PVDF) membranes for immunoblotting or lectin blotting after incubation with the appropriate primary antibodies (Abs for human LF, IgG, and IgA were purchased from Abcom) or biotin-conjugated AOL (Seikagaku, Tokyo, Japan) (23).

### Fecal DNA extraction, 16S rRNA, and metagenomic sequencing.

The microbial genome DNA from fecal samples of infants and mice was extracted using the E.Z.N.A. stool DNA kit (Omega Bio-tek, Inc.) according to the manufacturer’s instructions. The library construction, qualification, and sequencing were done by Illumina HiSeq (Novogene Bioinformatics Technology Co., Ltd., Beijing, China) (79); for details regarding 16S rRNA and metagenomic sequencing, please see Text S1.

### Mice.

Heterozygous *Fut8*^+/−^ mice on the ICR background were maintained in a room illuminated for 12 h (08:00 to 20:00 h) and kept at 24 ± 1°C with free access to food and water in the specific-pathogen-free laboratory animal facility of Dalian Medical University. All of the animal experiments were conducted according to the *Guide for the Care and Use of Laboratory Animals* (NIH publication no. 8023 [80]). Each *Fut8*^+/−^ and *Fut8^+/+^* maternal mouse nurtured three female neonates (*n* = 3).

### Flow cytometric analysis.

Thymus, spleen, and intestinal PPs of mice were picked up and ground in phosphate-buffered saline (PBS), and 1 × 10^6^ cells were used for flow cytometry with a fluorescence-activated cell sorter (FACS). Cells were incubated with anti-mouse CD16/CD32 monoclonal antibody (MAb) to block Fcγ receptors for 15 min and then stained on ice for 30 min with combinations of MAbs, as indicated in the figure legends. The MAbs used in this study are anti-mouse CD16/CD32 (2.4G2), phycoerythrin (PE)-conjugated Cy5-labeled anti-mouse CD4 (GK1.5), allophycocyanin (APC)-labeled anti-mouse CD8 (53-6.7), PE-labeled anti-mouse CD3 (2C11), PE-Cy5-labeled anti-mouse CD19 (6D5), fluorescein isothiocyanate (FITC)-labeled anti-mouse CD69 (H1.2F3), FITC-labeled anti-mouse Gr-1 (8C5), PE-labeled anti-mouse CD11c (N418), FITC-labeled anti-mouse pan-NK cells (DX5), PE-labeled anti-mouse F4/80 (BM8), and APC-labeled anti-mouse erythroid cells (TER119), all purchased from BD Biosciences. The flow cytometry assay was performed on a FACS-Calibur (Becton Dickinson, Mountain View, CA) and analyzed using FlowJo sofware (Tree Star).

### Synthesis of Fuc-α1,6-GlcNAc and Fuc-α1,6-GlcNAc-GlcNAc.

The synthetic route of Fuc-α1,6-GlcNAc-GlcNAc was described in our previous study (41). The synthetic route of the disaccharide Fuc-α1,6-GlcNAc (23) starts from the raw material of 1-β-azido-2-acetylamino-2-deoxy-3,4,6-*O*-triacetyl-d-glucopyranoside (compound 19). Compound 19 was dissolved in methanol and treated with NaOMe for 2 h at room temperature to give 1-β-azido-2-acetylamino-2-deoxy-d-glucopyranoside. The obtained product reacted with trityl chloride and pyridine to give 1-β-azido-2-acetylamino-2-deoxy-6-*O*-trityl-d-glucopyranoside. Then, it was treated with benzoyl chloride and pyridine to give 1-β-azido-2-acetylamino-2-deoxy-3,4-*O*-dibenzoyl-6-*O*-trityl-d-glucopyranoside (compound 20). Compound 20 was treated with AcOH-H_2_O to remove the 6-trityl and give compound 21. The glycosylation reaction of compound 21 and compound 14 was carried out in the presence of a sterically hindered base, 2,6-di-tert-butyl-pyridine, to give disaccharide 22. After debenzoylation with NaOMe and next-step reduction of the azido group, disaccharide 23 was created.

### Growth of strains in the presence of synthetic oligosaccharides.

The standard strains used in this study were obtained from China General Microbiological Culture Collection Center (CGMCC). They were routinely grown at 37°C on MRS medium supplemented with 0.1% l-cysteine and under anaerobic conditions. The strains were diluted to an optical density at 546 nm (OD_546_) of 0.1 in 1 ml of sugar-free MRS basal medium (73) containing 10 mM Fuc-α1,6-GlcNAc-GlcNAc or Fuc-α1,6-GlcNAc. Growth was monitored by measuring the OD_546_. Three technical replicates were performed for each strain.

### Cells and culture conditions.

The 3-83 B cells (expressing IgG2a-BCR recognizing p31) were purchased from the American Type Culture Collection. Cells were grown in RPMI 1640 supplemented with 2 mM glutamine, 50 mM 2-mercaptoethanal (ME) (Fluka, Buchs, Switzerland), 5% fetal calf serum (FCS), 100 U/ml penicillin, and 100 mg/ml streptomycin.

In order to detect the activation of B cells, approximately 1 × 10^6^ cells were cultured in serum-free medium for 12 h. Then, p31, 1,2-propanediol, and lactate were added to serum-free medium, respectively, at 1 and 10 μg/ml, for 5 min at room temperature. After that, cells were solubilized in lysis buffer (50 mM Tris-HCl [pH 8], 1% [vol/vol] Triton X-100, 150 mM NaCl, 10% [vol/vol] glycerol, 2 mM EDTA, 100 mM phenylmethylsulfonyl fluoride [PMSF], 5 mg/ml leupeptin, 1 mg/ml aprotinin, 100 mM NaF, and 1 mM sodium orthovanadate). Insoluble debris was removed after centrifugation at 10,000 × *g* for 10 min at 4°C. The same amounts of samples were separated by 10% SDS-PAGE and transferred to PVDF film. After blocking with 5% bovine serum albumin (BSA) for 1 h, the films were incubated in sequence with primary antibodies (Syk [D3Z1E] rabbit MAb, phospho-Syk [Tyr323] Ab, phospho-p44/42 MAPK [Erk1/2; Thr202/Tyr204] Ab, and p44/42 MAPK [Erk1/2] Ab, all purchased from Cell Signaling) overnight at 4°C and then with appropriate secondary antibodies (horseradish peroxidase [HRP]-conjugated goat anti-rabbit and anti-mouse IgG, purchased from Proteintech). Density analysis was performed using Quantity One software.

### Molecular interaction detection by Biacore.

BCR molecules were purified according to the method described in reference 41. The interactions between BCR and L-fucose metabolites were performed at room temperature using a BIAcoreT100 system with CM5 chips (GE Healthcare). An HBS-EP buffer consisting of 150 mM NaCl, 10 mM HEPES (pH 7.4), and 0.005% (vol/vol) Tween 20 was used as running buffer. The blank channel of the chip served as the negative control. To measure the interaction between L-fucose metabolites and BCR, 1 μg/ml BCR protein was immobilized on the chip. Gradient concentrations of lactate and 1,2-propanediol were then flowed over the chip surface. The binding kinetics were analyzed with the software BIA Evaluation version 4.1 using a 1:1 Langmuir-binding model (81).

### Statistical analysis.

The comparisons between two groups were performed using an unpaired Student's *t* test with Welch’s correction by Graph Pad Prism version 5 (Graph Pad Software, La Jolla, CA). The data are shown as mean ± standard error of the mean (SEM). *P* values of <0.05 are considered statistically significant (*, *P* < 0.05; **, *P* < 0.01; ***, *P* < 0.001). For gut microbiota analysis, the Chao1 index and the Shannon index at the genus level were calculated with QIIME (version 1.7.0). The abundance and diversity of the OTUs (beta diversity) were examined using principal-coordinate analysis (PCoA) with unweighted UniFrac analysis in R software. LEfSe was used with the Kruskal-Wallis rank sum test to detect features with significantly different abundances between assigned taxa, and linear discriminant analysis (LDA) was performed to estimate the effect size of each feature. Bacterial groups with an LDA score of ≧4.00 were shown as significantly abundant within the indicated group. Data correlating to differential abundances of KEGG modules and CAZy enzymes were tested by Wilcoxon’s rank sum test, and *P* values were corrected for multiple testing by the Benjamin and Hochberg method. The bacterial genes with an LDA score of ≧2.5 were shown as significantly abundant in the indicated group. Statistical analysis of the quantitative multiple-group comparisons was performed using one-way analysis of variance (ANOVA [and nonparametric test]), followed by Tukey’s test (to compare all pairs of columns) with the assistance of GraphPad Prism 5. When analyzing the growth of mice or bacteria, two-way ANOVA were performed with the assistance of GraphPad Prism 5.

### Data availability.

The sequencing data were deposited in NCBI SRA under the accession number PRJNA527059.
