# Supplementary figures and images for: Core Fucosylation of Maternal Milk N-Glycan Evokes B Cell Activation by Selectively Promoting the l-Fucose Metabolism of Gut Bifidobacterium spp. and Lactobacillus spp
Source: mBio. 2019 Apr 2;10(2):e00128-19. doi: 10.1128/mBio.00128-19 (PMC6445936; doi:10.1128/mBio.00128-19)

A

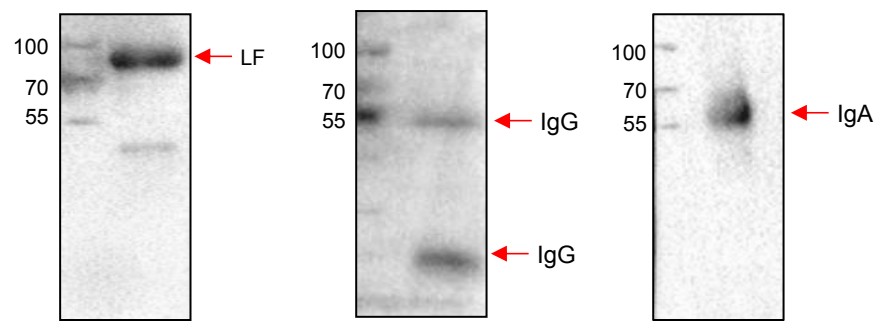

B

Day 6

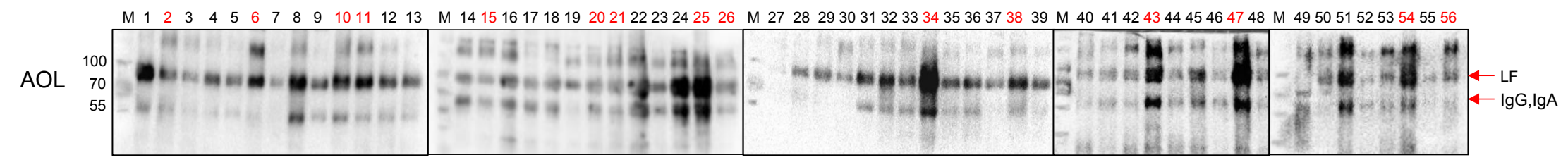

Day 42

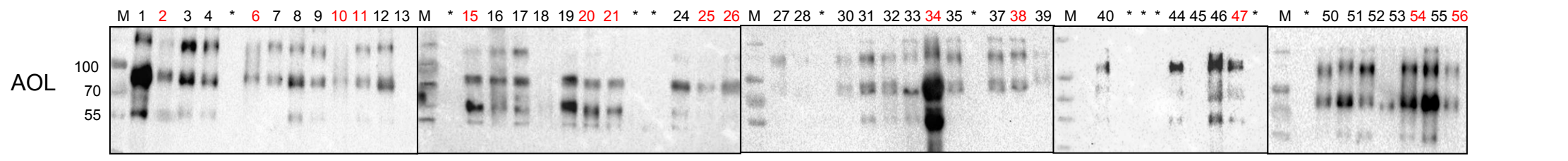

Day 120

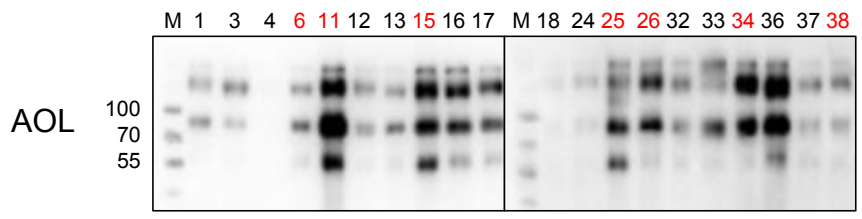

Supplement: FIG S1 [file mBio.00128-19-sf001.pdf]

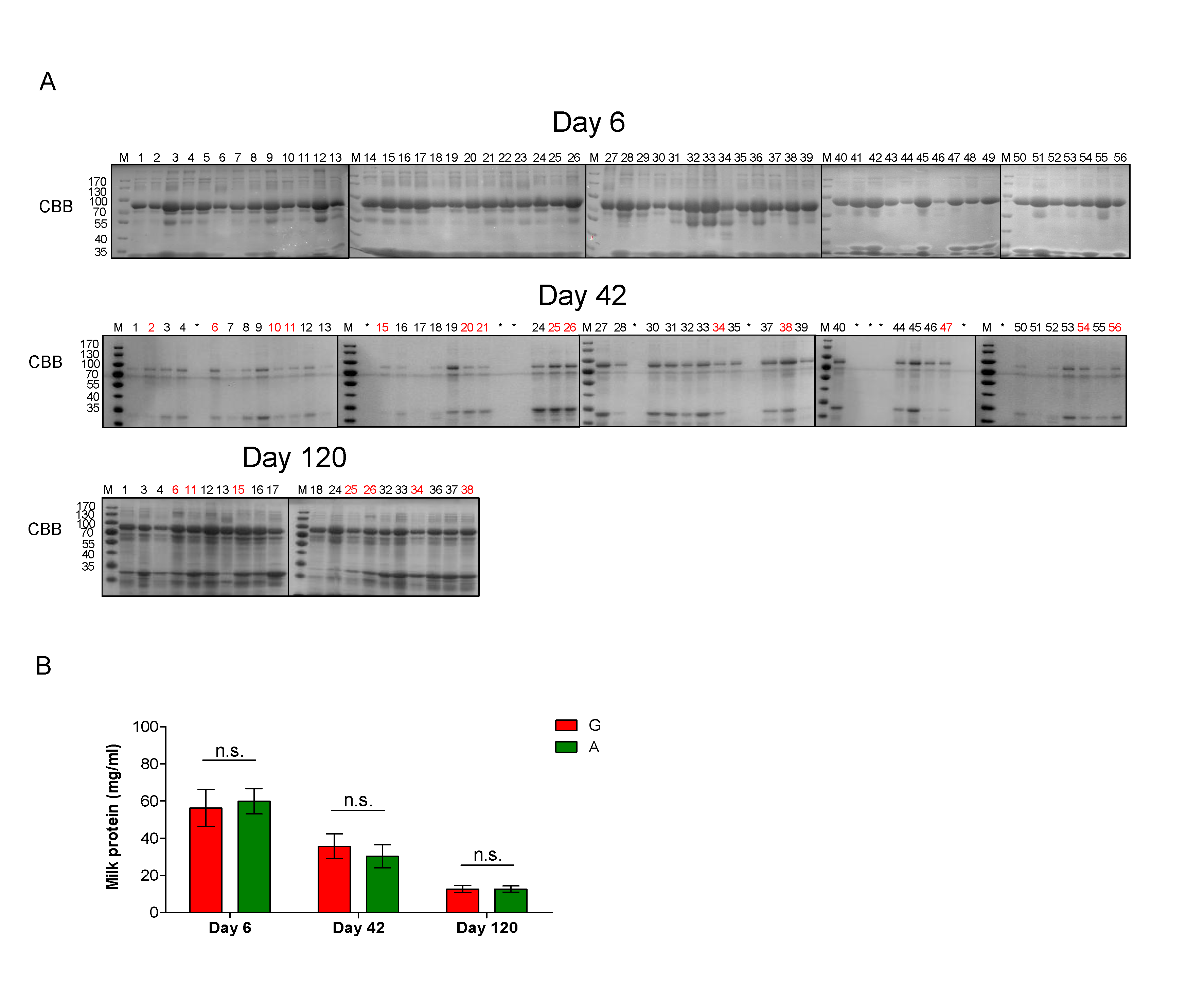

Supplement: FIG S2 [file mBio.00128-19-sf002.tif]

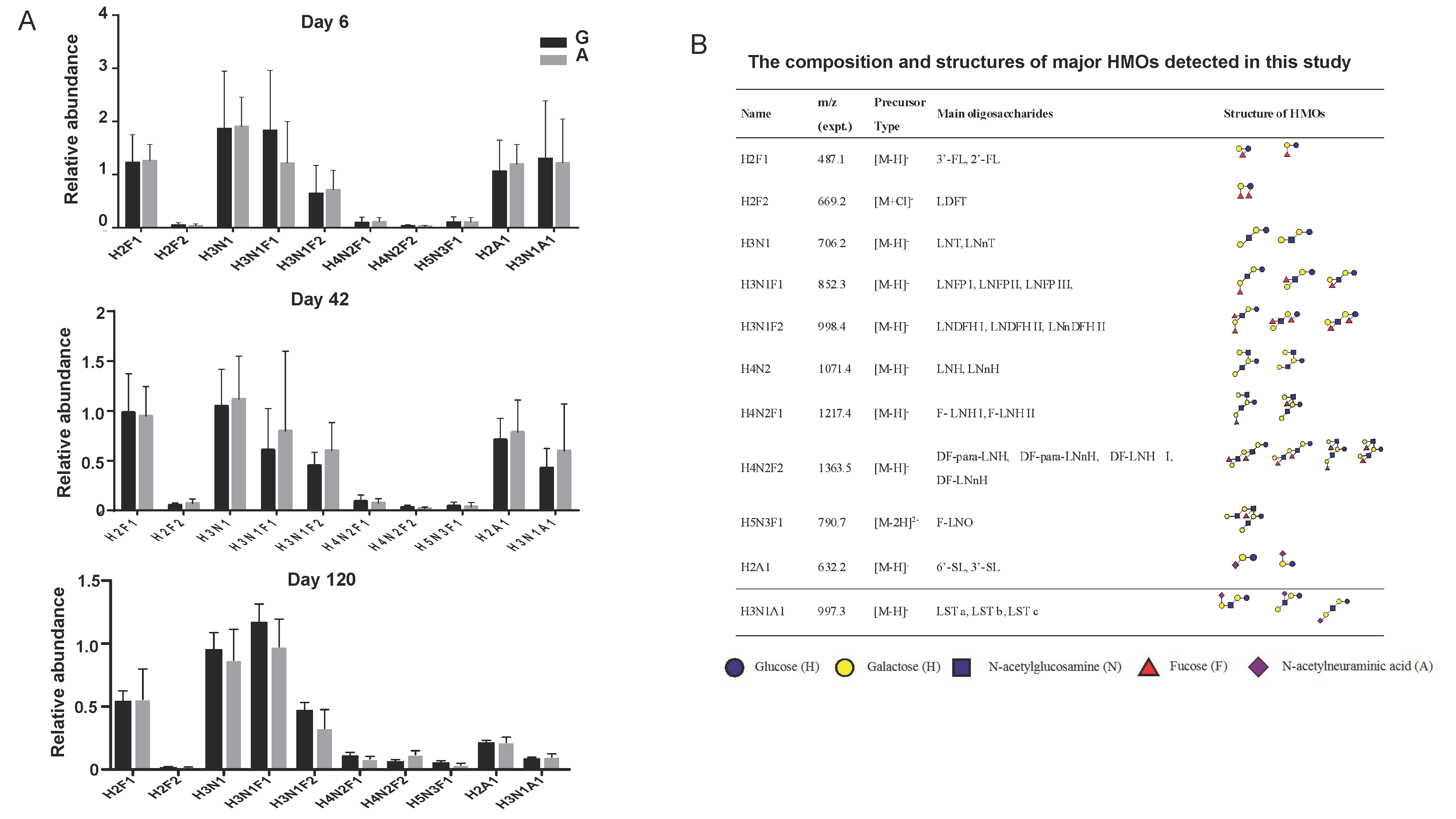

Supplement: FIG S3 [file mBio.00128-19-sf003.tif]

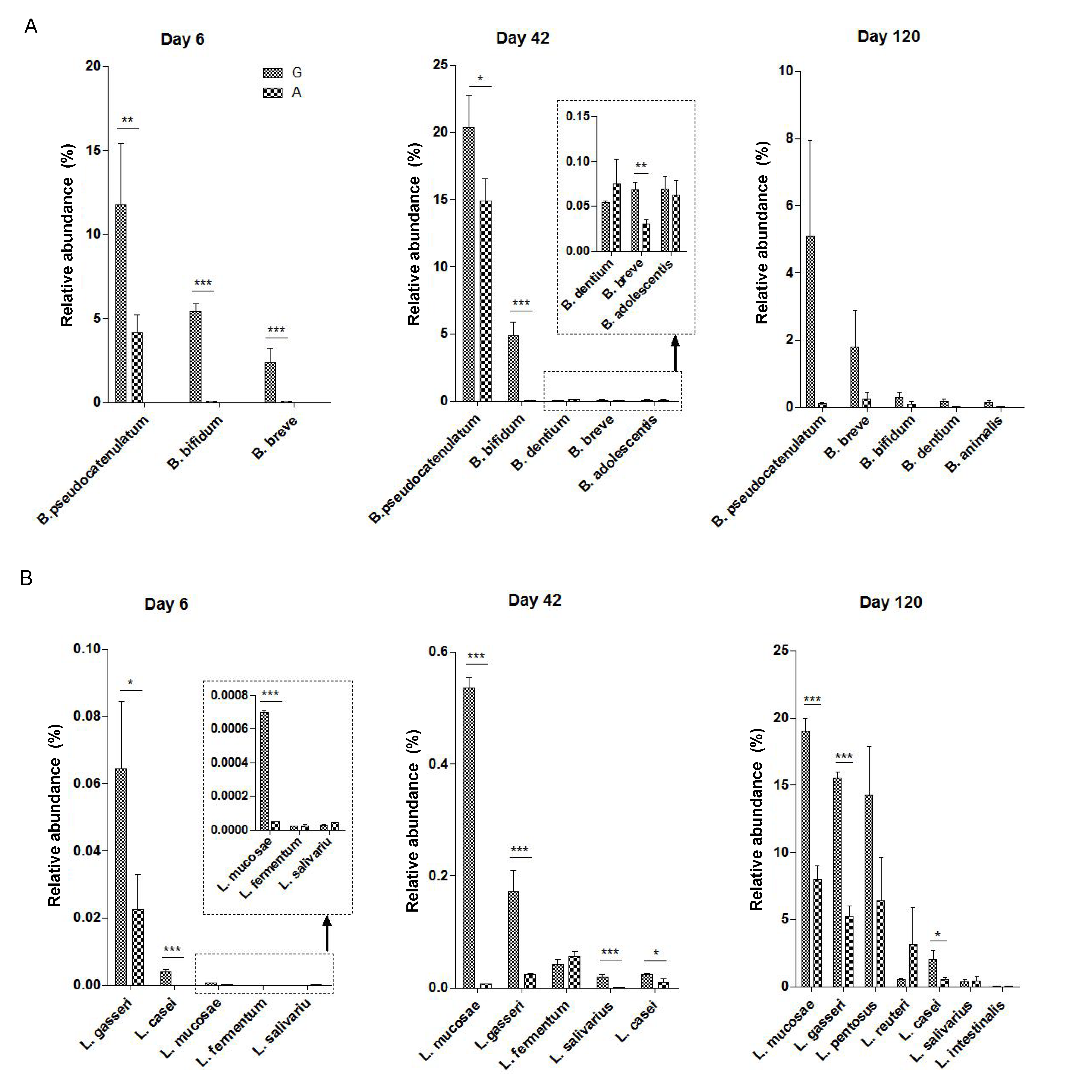

Supplement: FIG S4 [file mBio.00128-19-sf004.tif]

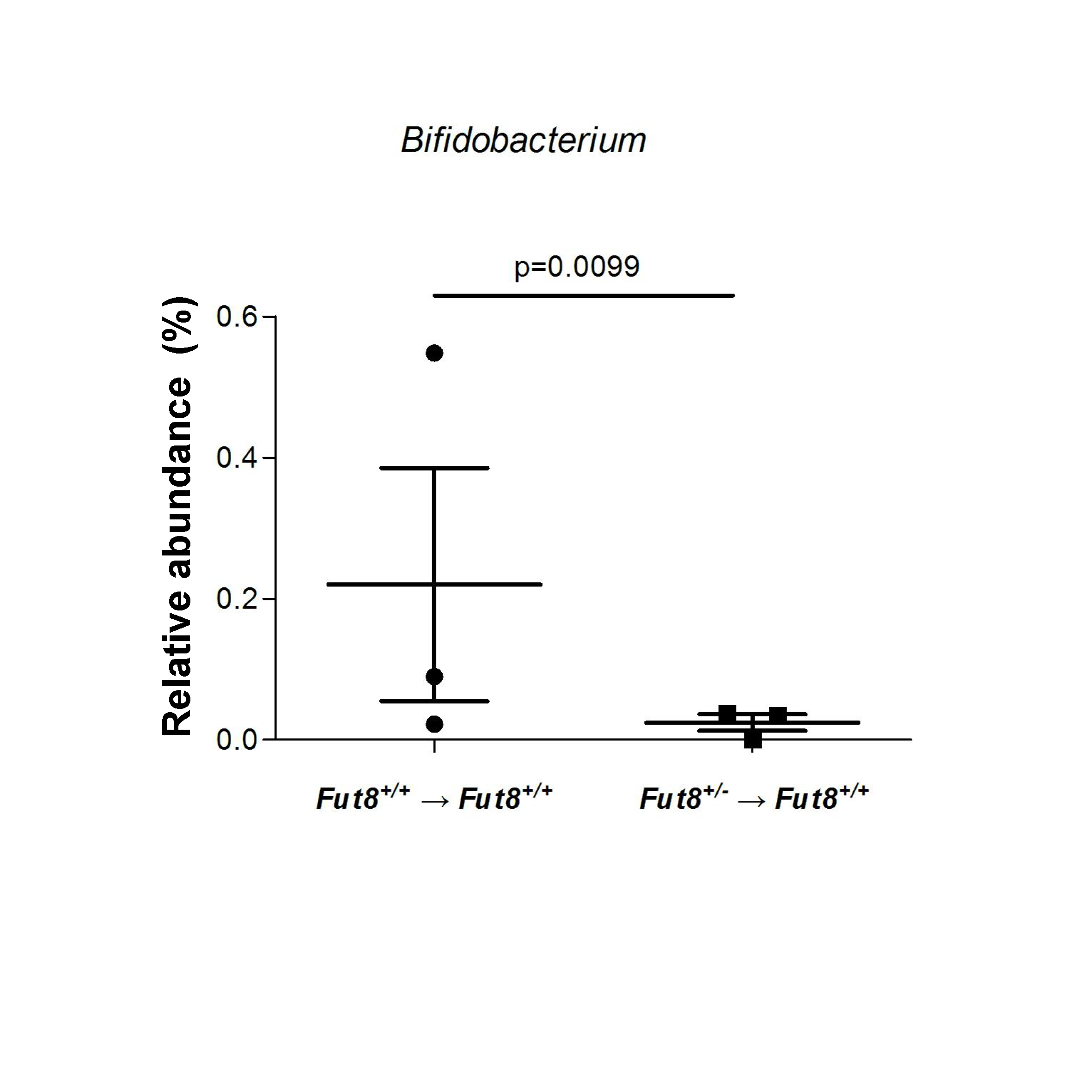

Supplement: FIG S5 [file mBio.00128-19-sf005.tif]

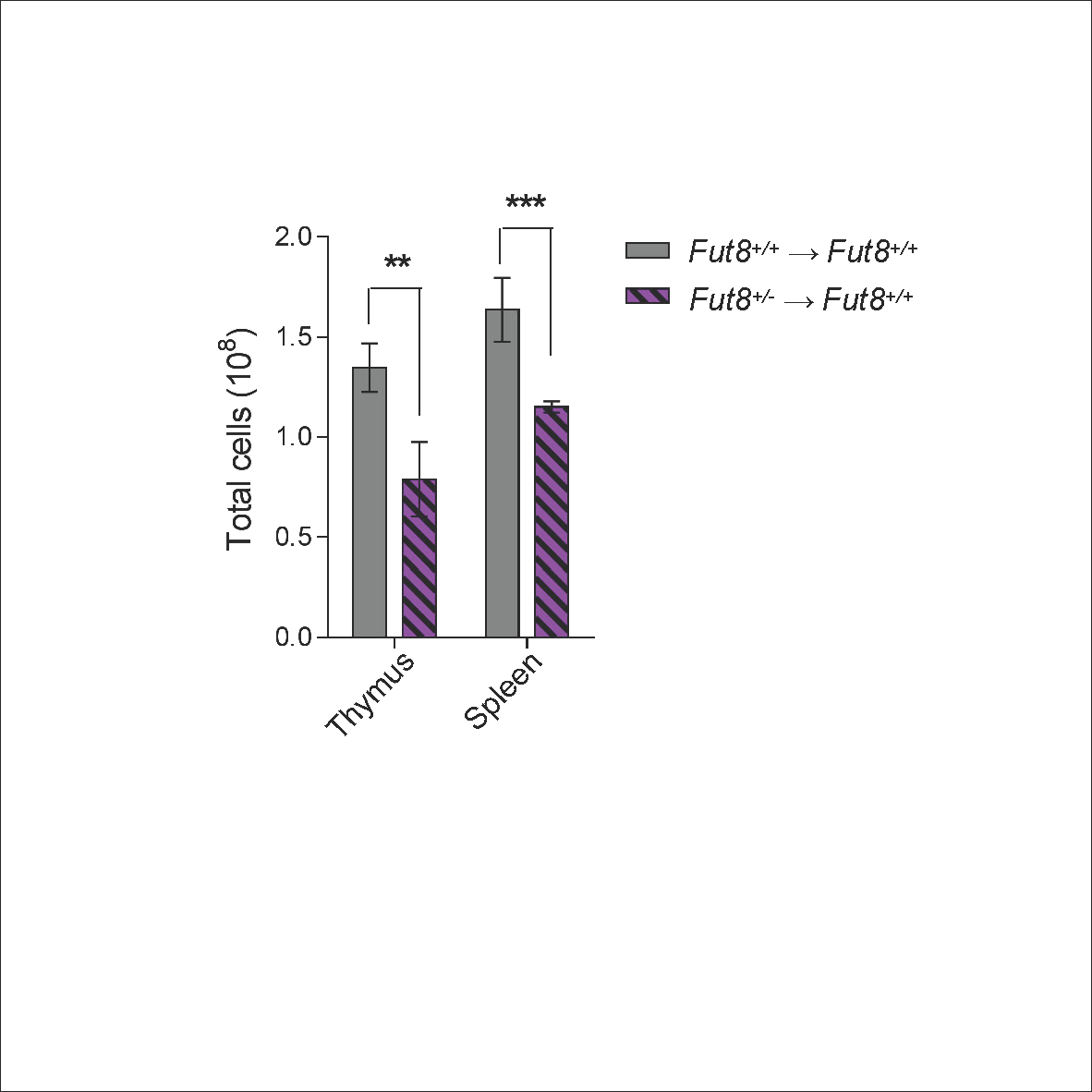

Supplement: FIG S6 [file mBio.00128-19-sf006.tif]
