# Supplementary material for: Core Fucosylation of Maternal Milk N-Glycan Evokes B Cell Activation by Selectively Promoting the l-Fucose Metabolism of Gut Bifidobacterium spp. and Lactobacillus spp
Source: mBio. 2019 Apr 2;10(2):e00128-19. doi: 10.1128/mBio.00128-19 (PMC6445936; doi:10.1128/mBio.00128-19)
Supplement: TABLE S1 [file mBio.00128-19-st001.docx]

**Supplementary Table S1 The polymorphism of *Fut8* gene at rs10483776, and the secretor status of different mothers enrolled in this study.**

| **Group** | **Samples** | **Sum** |
| --- | --- | --- |
| **G** | 2; 6; 10; 11; 15; 20; 21; 25; 26; 34; 38; 43; 47; 54; 56. | 15 |
| **A** | 1; 3; 4; 5; 7; 8; 9; 12; 13; 14; 16; 17; 18; 19; 22; 23; 24; 27; 28; 29; 30; 31; 32; 33; 35; 36; 37; 39; 40; 41 42; 44; 45; 46; 48; 49; 50; 51; 52; 53; 55. | 41 |
| **Secretor** | 1; 2; 4; 5; 6; 7; 11; 12; 13; 14; 15; 16; 17; 18; 19; 20; 22; 24; 26; 27; 29; 30; 32; 33; 34; 36; 37; 38; 39; 42; 43; 44; 45; 46; 47; 48; 49; 50; 51; 53; 54; 55; 56 | 43 |
| **Non-secretor** | 3; 8; 9; 10; 21; 23; 25; 28; 31; 35; 40; 41; 52 | 13 |
