# Supplementary material for: Core Fucosylation of Maternal Milk N-Glycan Evokes B Cell Activation by Selectively Promoting the l-Fucose Metabolism of Gut Bifidobacterium spp. and Lactobacillus spp
Source: mBio. 2019 Apr 2;10(2):e00128-19. doi: 10.1128/mBio.00128-19 (PMC6445936; doi:10.1128/mBio.00128-19)
Supplement: TABLE S2 [file mBio.00128-19-st002.docx]

**Supplementary Table S2 Comparison of alpha diversity indexes of infants’ gut microbiota between groups**

| **group** | **observed_species** | **shannon** | **simpson** | **chao1** | **ACE** | **goods_coverage** | **PD_whole_tree** |
| --- | --- | --- | --- | --- | --- | --- | --- |
| **A (Low)** | 122 | 2.415 | 0.66 | 143.676 | 147.022 | 0.999 | 12.798 |
| **G (High)** | 122 | 2.528 | 0.687 | 178.854 | 161.441 | 0.999 | 18.821 |
| **P value** | 0.9741 | 0.6448 | 0.6529 | 0.2386 | 0.4218 | 0.7615 | 0.0781 |
