# Supplementary material for: Core Fucosylation of Maternal Milk N-Glycan Evokes B Cell Activation by Selectively Promoting the l-Fucose Metabolism of Gut Bifidobacterium spp. and Lactobacillus spp
Source: mBio. 2019 Apr 2;10(2):e00128-19. doi: 10.1128/mBio.00128-19 (PMC6445936; doi:10.1128/mBio.00128-19)
Supplement: TABLE S3 [file mBio.00128-19-st003.docx]

**Supplementary Table S3 The bacterial strains used in this study**

| **Species** | **Strain** | **Culture collection** | **Culture medium for enrichment** | **Ref** |
| --- | --- | --- | --- | --- |
| *B. breve* | ATCC15700 | ATCC^a^ | Nutrient broth (1% glucose) 100.0 ml  Sterile horse serum 300.0 ml  Sterilize intermittenttly in coagulator | [1] |
| 1. *longum subsp.*   *infantis* | CGMCC1.5078 | CGMCC^b^ | Meat broth:  Meat trypsinized broth 500.0 ml  Beef infusion broth 500.0 ml  Glucose 5.0 g  Gelatin 4.0 g  Agar 15.0 g  pH 8.2  Crumbled beef (just the right amount in tube) | This study |
| *B. peudocatenulatum* | CGMCC1.5001 | CGMCC | Meat broth | This study |
| *L. gasseri* | ATCC33323 | ATCC | MRS:  Casein peptone 10.0 g  Beef extract 10.0 g  Yeast extract 5.0 g  Glucose 5.0 g  Sodium acetate 5.0 g  Diamine citrate 2.0 g  Tween 80 1.0 g  K_2_HPO_4_ 2.0 g  MgSO_4_.7H_2_O 0.2 g  MnSO_4_.H_2_O 0.05 g  CaCO_3_ 20.0 g  Agar 13.0 g  Distilled water 1000 ml  PH 6.8 | [2] |
| *L. casei* | ATCC334 | ATCC | MRS | [3] |
| *L. brevis* | DM9218 ^c^  (CGMCC1.2797) | CGMCC | MRS | [4] |

^a^ATCC: American Type Culture Collection.

^b^CGMCC: China General Microbiolgical Culture Collection Center.

^c^The strain *L. brevis* DM9218 was isolated from fermented Chinese food by our lab [6], and it was deposited in CGMCC (CGMCC1.2797).

1. Van Beek AA, Hoogerland JA, Belzer C, De Vos P, De Vos WM, Savelkoul HF, Leenen PJ. 2016. Interaction of mouse splenocytes and macrophages with bacterial strains in vitro: the effect of age in the immune response. Benef Microbes 7(2):275-87.
2. Honda H, Nagaoka S, Kawai Y, Kemperman R, Kok J, Yamazaki Y, Tateno Y, Kitazawa H, Saito T. 2012. Purification and characterization of two phospho-β-galactosidases, LacG1 and LacG2, from Lactobacillus gasseri ATCC33323(T). J Gen Appl Microbiol.58(1):11-7.
3. Bidart GN, Rodriguez-Diaz J, Palomino-Schatzlein M, Monedero V, Yebra MJ. 2017. Human milk and mucosal lacto- and galacto-N-biose synthesis by transgalactosylation and their prebiotic potential in Lactobacillus species. Appl Microbiol Biotechnol 101:205-215.
4. Wang H, Mei L, DengY , Liu Y, Wei X, Liu M, Zhou J, Ma H, Zheng P, Yuan J, Li M. 2019. Lactobacillus brevis DM9218 ameliorates fructose-induced hyperuricemia through inosine degradation and manipulation of intestinal dysbiosis. Nutrition, doi: https://doi.org/10.1016/j.nut.2018.11.018.
