# Supplementary material for: Core Fucosylation of Maternal Milk N-Glycan Evokes B Cell Activation by Selectively Promoting the l-Fucose Metabolism of Gut Bifidobacterium spp. and Lactobacillus spp
Source: mBio. 2019 Apr 2;10(2):e00128-19. doi: 10.1128/mBio.00128-19 (PMC6445936; doi:10.1128/mBio.00128-19)
Supplement: TEXT S1 [file mBio.00128-19-s0001.docx]

**Supplementary materials and methods**

**HMOs extraction and detection.** 200 μl of milk was centrifuged at 9,000 rpm for 20 min at 4 °C to remove lipid. Ethanol (400 μl) was then added to the skim milk before centrifugation at 9,000 rpm for 10 min at 4 °C to remove protein. The obtained supernatant was diluted 10-fold and used for analysis. The analysis of oligosaccharides was carried out on an LC-MS system, in which an Agilent 1290 series LC unit was coupled with an Agilent 6540 series time-of-flight mass spectrometry. The drying gas temperature was 350 °C with a flow rate of 8.0l/min. Both MS and MS/MS spectra were acquired in the negative-ion mode with an acquisition rate of 1 s per spectrum over a mass range of m/z 300-2000 (for MS) and m/z 50-2000 (for MS/MS). Precursor-ion selection was made automatically by the data system based on ion abundance. Three precursors were selected from each MS spectrum to carry out product-ion scanning. Collision energy of 30V was used for collision-induced dissociation (CID). HMO identification and quantification were performed via Agilent Mass Hunter Qualitative Analysis software (version B.03.01). A new column (named ASP, 150 mm×2.1 mm I.D.) was used as stationary phase. The mobile phase for separation of standard mixture was constituted with water and acetonitrile. The solvent gradient was performed at a flow rate of 0.2 ml/min as follows: 0-40 min, 20-50% water. The injection volume was 2 μl.

**Fecal DNA extraction, polymerase chain reaction (PCR) and 16S rRNA amplicon data processing.** The microbial genome DNA from fecal samples of infants was extracted using E.Z.N.A. ® Stool DNA kit (Omega Bio-tek, Inc.) according to the manufacturer's instructions. A Nanodrop 2000 spectrophotometer was used to evaluate the purity and concentration of isolated DNA. The universal primers (520F, 802R) were used to amplify the V4 region of 16S rDNA from metagenomic DNA in mice feces. Primer sets were modified with Illumina adapter regions for sequencing on the IlluminaGAIIx platform (Illumina, San Diego, CA, USA). The reverse primers were modified with an 8-bp Hamming error correcting barcode to distinguish among samples. The 50 μl PCR mixture contained the following components: 100 ng of DNA template, 5 μl PCR buffer, 1 μl dNTPs, 0.25 μl HotStarTaq® Plus DNA Polymerase (Qiagen), and 2.5 pmol of each primer. The PCR program consisted of an initial step at 95 °C for 5 min; 30 cycles of 94 °C for 45 s, 55 °C for 45 s, and 72 °C for 60 s; and a final extension at 72 °C for 8 min. PCR products were checked by 1.5% (w/v) agarose gel electrophoresis in 0.5 mg/ml ethidium bromide and purified with the Qiaquick gel extraction kit (Qiagen). Sequences of 16S rDNA were detected by Illumina HiSeq (reconstructed cDNA sequence: 2×250 bp, Novogene Bioinformatics Technology Co. Ltd, Beijing). The sequences obtained after quality control analysis were used in the present analysis, which were uploaded to QIIME (Quantitative Insights Into Microbial Ecology, v1.8.0) for further study. The operational taxonomy units (OTUs) of representative sequences at a similarity cutoff of 97% and their relative abundance (alpha-diversity) were used to calculate Shannon and other indexes by UCLUST. The abundance and diversity of the OTUs (beta-diversity) were examined using Principal coordinates analysis (PCoA) with unweighted UniFrac analysis in R software. The statistical significance of the separation among groups was assessed by the linear discriminant analysis effect size (LEfSe) method based on linear discriminant analysis scores exploited by Curtis Huttenhower (http://huttenhower.sph.harvard.edu/galaxy/), which used the nonparametric factorial Kruskal–Wallis and Wilcoxon rank sum test to identify key OTUs for separating different treatment groups at a significance level of 0.05. This work was conducted by Levelgene Bio-Pharm Technology Co., Ltd, Dalian, China.

**Metagenomic sequencing and gene catalogue construction.** The qualified DNA samples taken from infant feces on day 42 post birth were randomly broken into fragments of about 350 bp by Covaris sonicator, and the whole library was prepared by terminal repair, A tail, sequencing ligation, purification, and PCR amplification. After the library was constructed, preliminary quantification was performed using Qubit 2.0, and the library was diluted to 2 ng/µl. Then, the insert size of the library was detected using Agilent 2100. After the insert size was as expected, the effective concentration of the library was determined by Q-PCR. Accurate quantification (effective library concentration > 3 nM) to ensure library quality. After the library was qualified, the different libraries were pooled according to the effective concentration and the target data volume, and sequenced by Illumina HiSeq (Novogene Bioinformatics Technology Co. Ltd, Beijing).

The raw data obtained by sequencing will have a certain proportion of low-quality data. In order to ensure the accuracy and reliability of the subsequent information analysis results, the raw data should first be quality-controlled and host-filtered. (1) Remove the low-quality bases(mass value ≤ 38) exceeds a certain ratio (default is 40bp) of reads; (2) remove N bases to a certain proportion of reads (default is set to 10bp); (3) remove the overlap between the adapter and the Adapter exceeds a certain threshold (The default is set to 15bp) of reads; (4) If the sample is host contaminated, it needs to be compared with the host database, and the filtering may be from the host (the default is SoapAligner software, parameter setting: identity ≥ 90%, -l 30, - v 7, -M 4, -m 200, -x 400) reads; get valid data (Clean Data). After pre-processing, Clean Data was obtained, and SOAP denovo assembly software was used for Assembly Analysis, and each sample was not put together for reading and mixing, in order to find low-abundance species information in the sample. Starting from each sample and mixed-assembled Scaftigs (>=500bp) the ORF (Open Reading Frame) prediction was performed using MetaGeneMark, and the information with a length less than 100 nt was filtered out from the prediction results. The ORF prediction results for each sample and mixed assembly were de-redundated using CD-HIT software to obtain a non-redundant initial gene catalogue, which was clustered by identity 95%, coverage 90% by default, and selected the longest sequence. For representative sequences, the parameters are: -c 0.95, -G 0, -aS 0.9, -g 1, -d 0. The genes supporting the number of reads <=2 in each sample were filtered out to obtain the gene catalogue (Unigenes) which was finally used for subsequent analysis. Alignment of Unigenes with Bacteria, Fungi, Archaea, and Viruses sequences from NCBI's NR (Version: 2016-11-05) database using DIAMOND software (blastp), evalue ≤ 1e-5). For the comparison of each sequence, select the comparison result of e value <= minimum evalue*10 for subsequent analysis. After filtering, since each sequence may have multiple alignment results, multiple different species classification information is obtained. In order to ensure its biological significance, the LCA algorithm (system classification applied to MEGAN software) is adopted, and the first branch will appear.
